# Soil microbial legacies differ following drying-rewetting and freezing-thawing cycles

**DOI:** 10.1038/s41396-020-00844-3

**Published:** 2021-01-06

**Authors:** Annelein Meisner, Basten L. Snoek, Joseph Nesme, Elizabeth Dent, Samuel Jacquiod, Aimée T. Classen, Anders Priemé

**Affiliations:** 1 Microbial Ecology, Department of Biology, Lund University, Ecology Building, SE-223 62 Lund, Sweden; 2 Department of Biology, University of Copenhagen, Universitetsparken 15, 2100 Copenhagen, Denmark; 3 Department of Microbial Ecology, Netherlands Institute of Ecology, Droevendaalsesteeg 10, Wageningen, The Netherlands; 4 Theoretical Biology and Bioinformatics, Utrecht University, 3584 CH Utrecht, The Netherlands; 5 Agroécologie, AgroSup Dijon, INRAE Centre Dijon, Université de Bourgogne Franche-Comté, Dijon, France; 6 Ecology and Evolutionary Biology Department, University of Michigan, Ann Arbor, MI 48109 USA; 7 The Gund Institute for Environment, University of Vermont, Burlington, VT USA; 8 The Center for Macroecology, Evolution and Climate, The University of Copenhagen, Copenhagen Ø, Denmark; 9 Department of Geosciences and Natural Resource Management, Center for Permafrost (CENPERM), University of Copenhagen, Øster Voldgade 10, 1350 Copenhagen, Denmark; 10 Present Address: Wageningen University & Research, Droevendaalsesteeg 4, Wageningen, The Netherlands

**Keywords:** Soil microbiology, Biogeochemistry, Biodiversity, Microbial ecology

## Abstract

Climate change alters frequencies and intensities of soil drying-rewetting and freezing-thawing cycles. These fluctuations affect soil water availability, a crucial driver of soil microbial activity. While these fluctuations are leaving imprints on soil microbiome structures, the question remains if the legacy of one type of weather fluctuation (e.g., drying-rewetting) affects the community response to the other (e.g., freezing-thawing). As both phenomenons give similar water availability fluctuations, we hypothesized that freezing-thawing and drying-rewetting cycles have similar effects on the soil microbiome. We tested this hypothesis by establishing targeted microcosm experiments. We created a legacy by exposing soil samples to a freezing-thawing or drying-rewetting cycle (phase 1), followed by an additional drying-rewetting or freezing-thawing cycle (phase 2). We measured soil respiration and analyzed soil microbiome structures. Across experiments, larger CO_2_ pulses and changes in microbiome structures were observed after rewetting than thawing. Drying-rewetting legacy affected the microbiome and CO_2_ emissions upon the following freezing-thawing cycle. Conversely, freezing-thawing legacy did not affect the microbial response to the drying-rewetting cycle. Our results suggest that drying-rewetting cycles have stronger effects on soil microbial communities and CO_2_ production than freezing-thawing cycles and that this pattern is mediated by sustained changes in soil microbiome structures.

## Introduction

Climatic change leads to more variable weather conditions characterized by extreme drought and rainfall fluctuations [1, 2] as well as altered frequencies of freezing-thawing cycles in temperate ecosystems [3]. Freezing-thawing and drying-rewetting fluctuations affect the state of water in soils and its bioavailability. In dry soil, water is replaced by air, whereas in frozen soil water becomes ice [4]. Upon rewetting, water availability increases *via* precipitation or flooding. Upon thawing, water availability increases *via* ice melting. Soil microorganisms have developed several strategies to adapt and face the ever-changing availability of water [5, 6] and heavily rely on soil moisture for many vital aspects, including mobility, feeding, and reproduction. Therefore, water availability is the primary factor affecting both soil microbiome structures and their activities [5, 6]. Thus, shifts in soil moisture availability will have important consequences on the soil communities that regulate ecosystem functions.

Microbial responses to freezing-thawing cycles are generally studied in sub-arctic climates [7, 8], while drying-rewetting cycles are generally studied in arid climates [9, 10]; yet, microorganisms in both environments appear to have similar responses to drying-rewetting and freezing-thawing cycles. For both environments, upon rewetting and thawing, a CO_2_ pulse is released from soil [4, 11, 12], which is likely a carbon cost to the soil as the pulsed carbon is respired away [7, 13, 14]. In addition, drying-rewetting affects the microbiome composition [15] and changes may persist for weeks to months after the ecosystem appears to have recovered, which is often referred to as a legacy [16–18]. Similarly, in cold adapted ecosystems, freezing-thawing cycles affect the microbiome composition [19, 20]. Clearly, drying-rewetting and freezing-thawing cycles in divergent ecosystems seem to impact soil communities in similar ways. Yet, the question remains if soil microorganisms living in the same ecosystem, will respond similarly to these two types of fluctuations, and if the order of events matters.

Exposure to environmental fluctuations may create a taxonomic legacy in the soil microbiome composition [16]. Future microbiome responses to fluctuations may be dependent on this taxonomic legacy. For example, soils with a history of drought contain more resistant bacteria when exposed to an additional drying-rewetting cycle [21]. A previous drought may affect the microbial responses to current moisture conditions [22–24] and multiple stressors appear to increase microbial recovery to future fluctuations [25–28]. A fluctuation can affect the microbial response to an additional, different fluctuation, such as extreme freezing following an extreme heat treatment [29]. Further, the order in which the extreme fluctuations occurs matters for soil microorganisms [30].

Soil microorganisms can be sensitive, tolerant, or opportunistic to drying-rewetting and freezing-thawing cycles [10, 31–33]. These different responses are contingent on the environmental niche of the individual microorganism to drying-rewetting and freezing-thawing [31, 34]. Sensitive microorganisms are damaged during drought or freezing [35, 36]. Recovery of these sensitive microorganisms is difficult and because water is limiting [37], they will likely be inactive [13, 38–40]. Microorganisms with desiccation resistance traits can remain active and produce biomass at subzero temperatures [41] or during water limitation. Microorganisms can also enter a dormant state until more favorable conditions occur [42, 43]. Traits that enable survival are important because surviving microorganisms can take advantage of empty microhabitat spaces and increased availability of substrates resulting from necromass [44, 45]. Fast responding and opportunistic microorganisms can colonize empty spaces first [46]. These early colonizers benefit from a “priority effect” and influence the chronology of the ensuing microbial species to rebound or invade spaces [47, 48]. All these microbial response patterns determine the community composition after one or more environmental fluctuations [16, 17].

Bacteria and fungi have different traits and responses to freezing-thawing and drying-rewetting cycles [33, 49]. Multicellular fungi are often more drought tolerant than bacteria [15, 17] due to their multicellular hyphal networks allowing remote access and redistribution of water along hydrological gradients within soil [50]. In contrast, fungi are more vulnerable than bacteria to freezing [51–53] and freeze-thaw cycles [33, 54]. As such, the community assembly of soil fungi and bacteria may respond differently to drying-rewetting and freezing-thawing cycles. However, little is known about how one type of weather fluctuation (e.g., drying-rewetting) affects the response of the soil microbiome to the other type of fluctuation (e.g., freezing-thawing).

The overall aim of this study was to test if drying-rewetting and freezing-thawing cycles leave a similar or different legacy in the soil microbiome. More specifically, we tested the hypothesis that a history of either one drying-rewetting or one freezing-thawing cycle makes the microbiome more tolerant to an additional fluctuation, be it the same fluctuation or not. In addition, we tested the hypothesis that fungi are sensitive to freezing-thawing cycles, but not to drying-rewetting cycles. To test these hypotheses, we set up three experiments. In the first, we tested if the soil microbiome structure and functioning would be similarly affected upon one drying-rewetting or freezing-thawing cycle (H1: the “similarity hypothesis”). In the second, we studied if the legacy of a freezing-thawing cycle influences the response of the soil microbiome to an additional drying-rewetting cycle compared to a control drying-rewetting cycle legacy (H2: the “freezing-thawing dependence hypothesis”). In the third experiment, we conversely studied if the legacy of a drying-rewetting cycle influences the response of the soil microbiome to an additional freezing-thawing cycle compared to a control freezing-thawing cycle legacy (H3: the “drying-rewetting dependence hypothesis”).

As DNA-based methods can reflect changes of active, inactive and dead microorganisms (e.g., relic DNA) [55, 56] we also investigated the potentially active (rRNA-based) fraction of the soil microbiome [57]. We performed cDNA- (from rRNA) and DNA-based amplicon sequencing of bacterial and fungal marker genes [58] and linked these data to respiration.

## Material and methods

### Soil

Soil was sampled on 27 July 2016 from five plots at a naturalized grassland in the Netherlands (52.03N, 5.45E) where agricultural production stopped in 1995 [59]. Four 1 kg soil samples were collected at each plot from the top 10 cm, sieved at 2 mm, homogenized per plot and stored at 5 °C until use. Soil was a loamy sand (clay 1.25%; silt 11.72 %; sand 87.02%), had 5.38 ± 0.13% SOM (as determined by LOI) and a pH of 5.29 ± 0.21.

We chose this soil, because data was available that confirmand it had experienced drought and freezing in the past. In the period 1981–2016, the mean air temperature was 10.3 °C and the mean yearly rainfall was 895 mm (measured ca. 40 km from the sampled location, www.knmi.nl). During these years, there were 108 periods of 9 consecutive dry days, averaging three dry spells per year. The longest dry period was 32 days in April 2007. At 5 cm depth, the soil experienced on average 7 days below 0 °C annually and the lowest minimum temperature was −6 °C in 1996 (KNMI, De Bilt, the Netherlands).

### Microcosm experiments

**Fig. 1:**
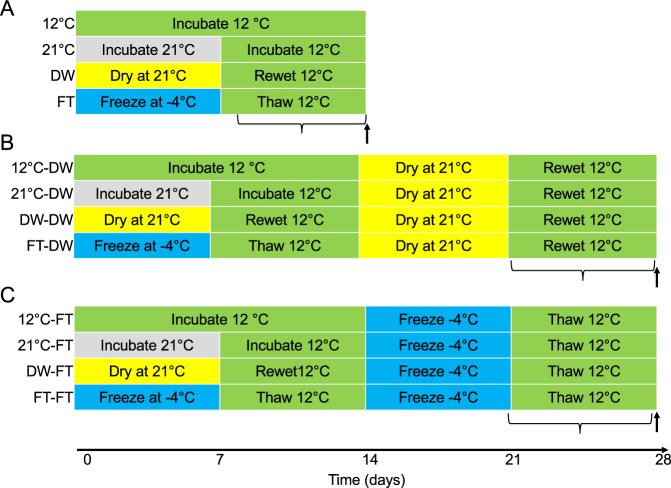
Experimental design. In the first experiment (**A**), the effect of a drying-rewetting or freezing-thawing cycle on greenhouse gas emission and microbial communities were studied. In the second experiment (**B**) the legacy effect of a freezing-thawing cycle on a drying-rewetting cycle was studied. In the third experiment (**C**), the legacy effect of a drying-rewetting cycle on a freezing-thawing cycle was studied. CO_2_ release was measured for 1 week following the last rewetting or thawing (indicated by the bottom-faced curly bracket), and RNA and DNA were isolated from the soil samples 1 week after rewetting or thawing (arrow).

We performed three experiments where each of the five soil samples were considered as independent blocks (Fig. 1). For each experiment, we set up one series of 20 166 ml PVC microcosms that contained 30 g field moist soil adjusted to 50% of water holding capacity (WHC). The experiments were initiated simultaneously.

### Experiment 1: Effect of drying-rewetting and freezing-thawing

The microcosms were pre-incubated at 12 °C for 1 month. Then, the soil was exposed to the following four treatments for 1 week: moist soil incubated at 12 °C, moist soil incubated at −4 °C (frozen), moist soil incubated at 21 °C, or soil incubated at 21 °C without lid (dry). After 1 week, all microcosms were incubated at 12 °C and the moisture content was adjusted to 50% WHC (Fig. 1A). Microcosms were sampled for greenhouse gas emissions and soil was snap-frozen with liquid nitrogen for RNA and DNA isolation (see below).

### Experiment 2: Legacy of drying-rewetting on freezing-thawing

In the second experiment (Fig. 1B), 20 additional microcosms with the four legacies were exposed to an additional drying-rewetting cycle. Greenhouse gas emissions were measured upon rewetting. Soil samples were snap-frozen with liquid nitrogen 1 week after rewetting.

### Experiment 3: Legacy of freezing-thawing on drying-rewetting

In the third experiment (Fig. 1C), 20 additional microcosms with the four legacies were analyzed as described for Experiment 2, except that these microcosms were exposed to an additional freezing-thawing cycle.

### Greenhouse gas measurements

Upon rewetting or thawing, 4 g soil samples from microcosms were placed in 54 ml respiration vials, aired with pressurized air, sealed with rubber septa [13] and incubated at 12 °C in darkness. Every 24–48 h, gas samples were extracted for analysis of CO_2_, CH_4_ and N_2_O on an SRI310C GC (SRI Instruments, Torrance, CA, USA) equipped with a Hayesep Q 80/100 column, an FID (connected to a methanizer) and an ECD. For Experiment 1, gas samples were taken at 26, 57, 98, 142 and 166 h post-rewetting or thawing. For Experiments 2 and 3, gas samples were taken at 22, 47, 72, 111, 140, 166 h. All measurement points were used to calculate cumulative gas production over 1 week.

### Nucleic acid isolation

Two grams of soil were snap-frozen with liquid nitrogen 1 week after rewetting or thawing and stored at −80 °C. RNA was isolated using the RNA PowerSoil^®^ Total RNA Isolation Kit according to the instructions of the manufacturer (Qiagen, Vedbæk, Denmark). DNA was removed from the isolated RNA using the DNaseMax Kit (Qiagen) with an extension of the DNase reaction to 1 h to ensure that all DNA was removed. RNA was reverse transcribed using RevertAid RT Reverse Transcription Kit (Thermo Fischer Scientific, Roskilde, Denmark) with random hexamer primers. DNA was co-isolated using RNA PowerSoil^®^ DNA Elution Accessory Kit (Qiagen). The quantity of isolated RNA and DNA was determined on a Qubit Fluorometer (Thermo Fischer Scientific), and the quality was determined on a 1% agarose gel. DNA contamination of the isolated RNA was checked using qPCR (see later for conditions). Isolation of RNA and DNA failed for one sample (see Tables S1–S4). This sample was excluded from further analysis.

### Quantification and sequencing of marker genes for prokaryotes and fungi

Quantitative PCR (qPCR) was performed on a Lightcycler^®^ 96 Real-Time PCR system (Roche, Hvidovre, Denmark). All qPCR reactions were run in technical duplicates. Prokaryote abundance was estimated by quantifying the V3 region of 16S rRNA gene as described previously [60] and fungal abundance by quantifying the ITS2 region as described previously [61].

To determine the composition of prokaryotic and fungal communities, sequencing of 16S rRNA genes and ITS2 fragments (DNA) and transcripts (cDNA) was done as described previously [62]. Raw sequence reads are deposited on NCBI Sequence Read Archive (https://www.ncbi.nlm.nih.gov/sra) under BioProject accession number PRJEB40946. Details of the qPCR protocol, the preparations for sequencing and annotation of sequence reads can be found in the supplementary information.

### Statistical analysis

Sequences were analyzed in R version 3.5.0 [63] using *phyloseq* and *vegan* [64, 65]. Sample summaries are presented in supplementary information (Table S1–S4). There were on average 16,525 16S cDNA reads (Table S1) and 5389 16S DNA reads per sample (Table S2). One 16S DNA sample was excluded due to low sequencing depth (Table S2). There were on average 60,355 ITS transcripts and 50,412 ITS DNA reads per sample (Tables S3–S4).

### Univariate statistics

For alpha-diversity analysis, samples were rarefied to equal depth before calculating richness and Shannon–Weaver diversity index. The rarefaction level was determined by the sample that had the lowest number of reads in each experiment (Tables S1–S4).

For all experiments, a similar univariate model was run to test differences in the response of the greenhouse gas emissions, abundance of 16S and ITS cDNA and DNA reads, Shannon–Weaver diversity index and OTU richness. Treatment was considered as a fixed factor and plot as random factor. We used the *LmerTest* and *lme4* packages [63, 66, 67]. A post-hoc test was run with the *diffslsmeans* function in the *LmerTest* package when there was a significant treatment effect [66].

### Multivariate analysis

Multivariate analysis was performed on non-rarefied samples. We performed a permutational multivariate analysis of variance on Bray–Curtis dissimilarity matrices to test treatment differences (*adonis* function, 10,000 permutations, strata = block) and a distance-based redundancy analysis on Bray–Curtis dissimilarity matrices (*capscale* function, 9999 permutations, strata = block).

### Heatmaps, OTU level analysis

For each category of 16S rRNA gene and ITS sequences (DNA and cDNA), only OTUs with a relative abundance of >0.25% in a sample were kept. Block effect was controlled for each OTU by subtraction of the OTU block mean (*adonis*, block effect *P* > 0.05). Treatment significance was determined by an ANOVA using each specific treatment as a factor. OTUs with a significant (ANOVA, *P* < 0.05) effect of the treatment were displayed in heatmaps to define response groups (*z*-score, *heatmap.2* function, *gplots* [68]). Taxonomic distribution in each response group was made using *ggplot2* [69].

### Networks and CO_2_ emissions

For each dataset (16S and ITS, DNA and cDNA), co-response networks were made using the relative abundance data, filtered for OTUs with >0.25% mean abundance. For each experiment, the Spearman’s rank correlation was calculated between OTUs. A coefficient cut-off of |0.65| was used for pairs to be taken up in the co-response network (approximate FDR for all categories = 0.02). To investigate the link with CO_2_ emission rates, the Spearman’s rank correlations between the relative abundance of each OTU and CO_2_ emission rates were calculated and used as a visualization overlay in the network figures. Network visualization was done in R using the packages, *ggplot2*, *igraph cowplot*, *extragrid* and *ggpubr*.

## Results

### Greenhouse gas emissions and microbial abundance

**Fig. 2:**
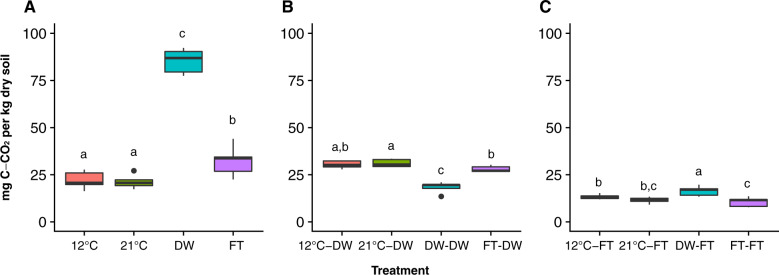
Cumulative CO_2_ released during a week upon rewetting and thawing. **A** Presents the cumulative respiration in Experiment 1, which tested the effect of drying-rewetting versus freezing-thawing. **B** Presents the cumulative respiration in Experiment 2, which tested the legacy of freezing-thawing on drying-rewetting. **C** Presents the cumulative respiration in Experiment 3, which tested the legacy of drying-rewetting on freezing-thawing. 12C indicates pre-incubation at 12 °C, while 21C indicates pre-incubation at 21 °C. DWindicates drying-rewetting cycle. FT indicates freezing-thawing cycle. Different superscript letters denote significant difference, post-hoc test at *P* < 0.05.

Both drying-rewetting and freezing-thawing treatments increased CO_2_ emission compared to controls (Figs. 2A; S1). One drying-rewetting cycle released approximately four times more CO_2_ than moist controls (at both 12 and 21 °C), while one freezing-thawing cycle released ~1.5 times more CO_2_ upon thawing than moist controls. A legacy of drying-rewetting decreased the release of CO_2_ upon a second drying-rewetting cycle compared with other legacies (Fig. 2B) whereas a legacy of drying-rewetting increased CO_2_ released upon the following freezing-thawing cycle (Fig. 2C). A legacy of freezing-thawing tended to decrease the release of CO_2_ after the second fluctuation (Fig. 2B, C).

Drying-rewetting and freezing-thawing cycles had only little effect on CH_4_ uptake or N_2_O emissions. There was no difference between treatments for CH_4_ uptake (Fig. S2). N_2_O emissions decreased when incubating the soil samples at 21 °C (Fig. S3). The abundance of 16S and ITS2 transcripts (cDNA) and genes (DNA) did not differ between treatments or legacies (Figs. S4, S5).

### Richness and Shannon–Weaver index

One drying-rewetting cycle decreased richness and Shannon–Weaver index in the 16S rRNA transcripts (cDNA; Figs. S6A, S7A) and this decrease remained present after a second drying-rewetting (Figs. S6C, S7C) or freezing-thawing cycle (Figs. S6E, S7E). In contrast, the history of one or two drying-rewetting or freezing-thawing cycles did not affect ITS2 transcripts (Figs. S6, S7). One drying-rewetting cycle increased 16S rRNA gene richness and evenness (DNA; Figs. S8A, S9A), and decreased richness and evenness of ITS2 and so did a freezing-thaw cycle (DNA; Figs. S8B, S9B). These changes in ITS2 richness and evenness were not observed after a second drying-rewetting or freezing-thawing cycle (DNA; Figs. S8C-F, S9C-F).

### Ordination

**Fig. 3:**
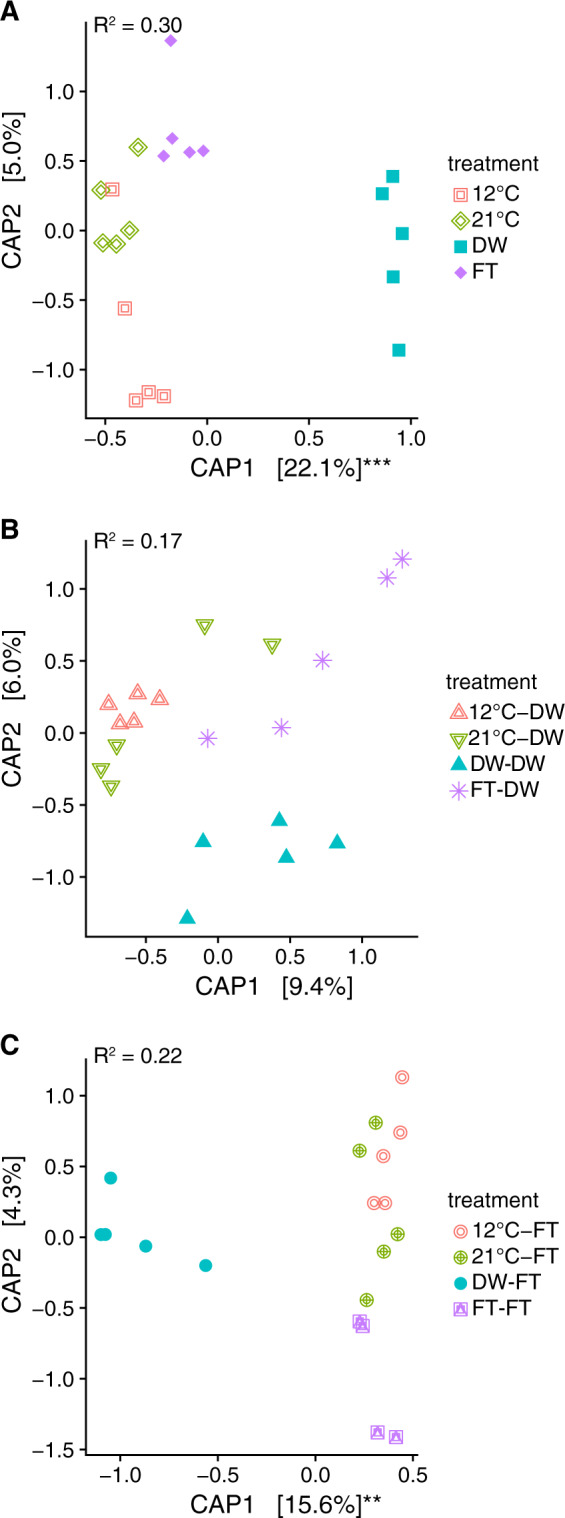
Partial distance-based redundancy analysis of prokaryotes for cDNA on Bray–Curtis dissimilarity using capscale ordination. **A** is Experiment 1 where we tested if a drying-rewetting (DW) or a freezing-thawing (FT) cycle leave different legacies in the prokaryote community. **B** is from Experiment 2 where we tested how the different legacies affected the microbial response to a drying-rewetting cycle. **C** is from Experiment 3 where we tested how different legacies affected the microbial communities after an additional ft cycle. 12C indicates pre-incubation at 12 °C, while 21C indicates pre-incubation at 21 °C (see Fig. 1). Significance of axes is tested with a permutation test by axis: **P < 0.01; ***p < 0.001.

One drying-rewetting cycle affected the 16S profiles, which remained different upon a freezing-thawing cycle, but not upon a second drying-rewetting cycle. The 16S profiles at cDNA level were separated in soils with a history of one drying-rewetting cycle (Fig. 3A; Table S5), but did not differ from each other when there were two drying-rewetting cycles or one freezing-thawing cycle prior to a drying-rewetting cycle (Fig. 3B; Table S5). In contrast, a drying-rewetting cycle prior to a freezing-thawing cycle separated the 16S cDNA profiles from the other legacies (Fig. 3C; Table S5). Similar results were observed for the 16S-DNA profiles (Fig. S10).

**Fig. 4:**
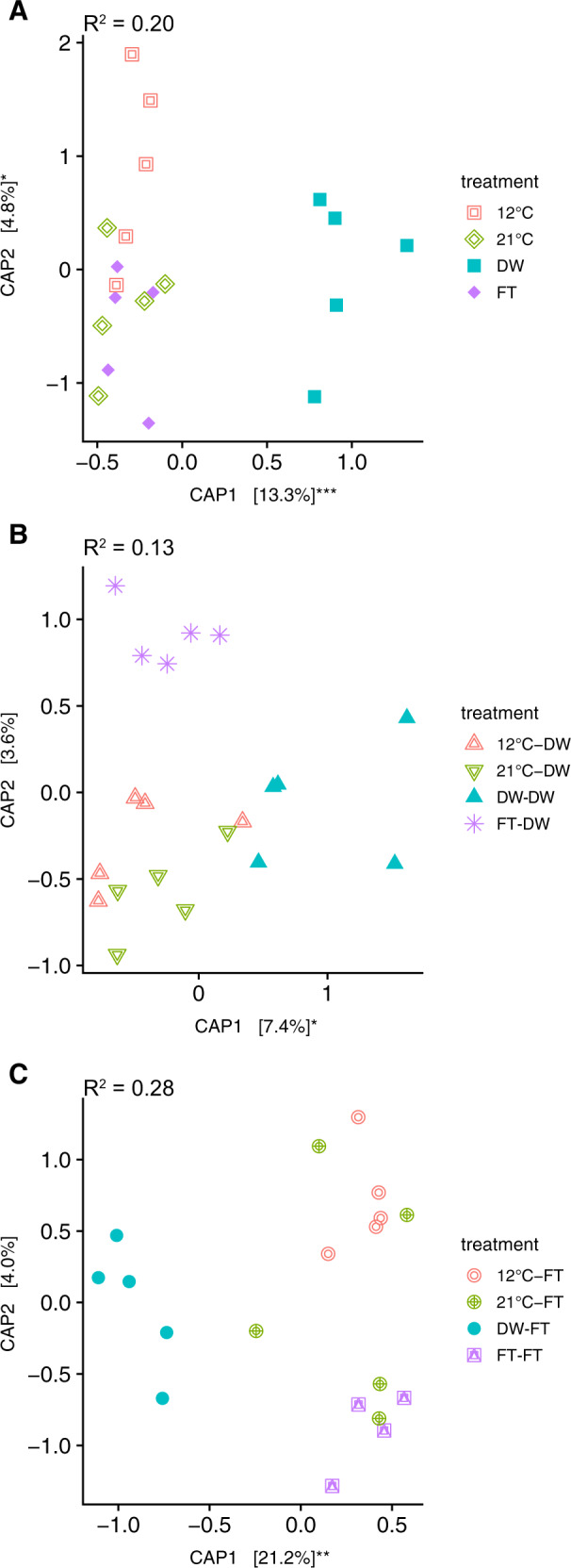
Distance-based redundancy analysis of ITS2 for cDNA on Bray–Curtis dissimilarity using capscale ordination. **A** is Experiment 1 where we tested if a drying-rewetting (DW) or a freezing-thawing (FT) cycle leave different legacies in the prokaryote community. **B** from Experiment 2 where we tested how the different legacies affected the microbial response to a drying-rewetting cycle. **C** is from Experiment 3 where we tested how different legacies affected the microbial communities after an additional ft cycle. 12C indicates pre-incubation at 12 °C, while 21C indicates pre-incubation at 21 °C (see Fig. 1). Significance of axes is tested with a permutation test by axis: *P < 0.05; **P < 0.01; ***P < 0.001.

The legacy of one drying-rewetting cycle also affected ITS2 profiles, remaining different upon a freezing-thawing cycle or a second drying-rewetting cycle. The ITS2-DNA and cDNA profiles were separated in soils with a history of one drying-rewetting cycle (Figs. 4A, S11A; Table S6) and these profiles remained different after two drying-rewetting cycles (Figs. 4B; S11B). In addition, a legacy of one drying-rewetting cycle affected the ITS2 profiles upon a freezing-thawing cycle (Figs. 4C, S11C, Table S6).

### Response groups within prokaryotes

**Fig. 5:**
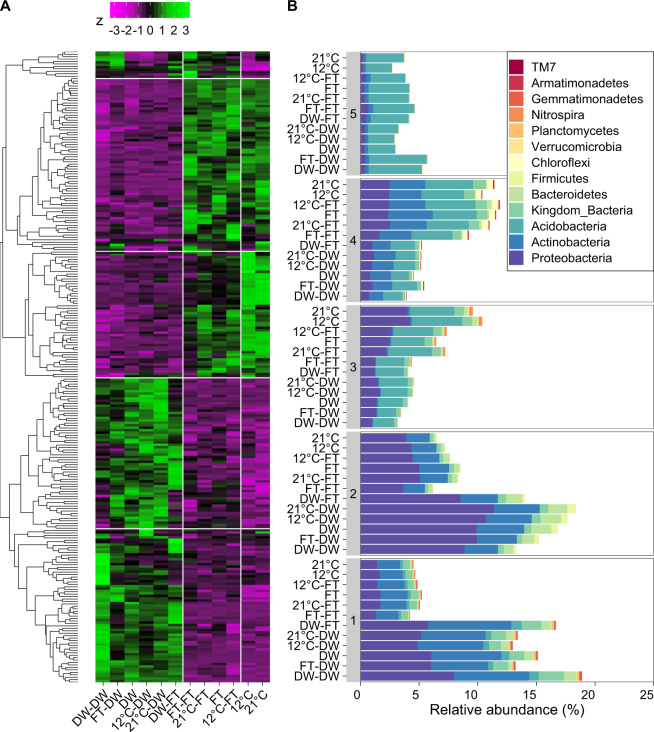
Response groups of 16S amplicons at cDNA level. **A** presents heatmap of the *z*-score per OTU per treatment compared to the mean relative abundance. **B** presents barplot with the relative abundance per phylum per response group. Response groups are labeled with numbers and correspond to the groups in (**A**), 1 is the bottom group, 5 is the top group, the rest is also shown in order.

Five response groups were identified for 16S-cDNA profiles, featuring 259 OTUs (Fig. 5; *P* < 0.05). In the first response group, OTUs were highest in relative abundance for samples with two drying-rewetting cycles, followed by samples with a single drying-rewetting cycle history; and OTUs without a history of drying-rewetting were lowest. In the second response group, OTUs were higher in samples with a history of one or two drying-rewetting cycles. In the third response group, OTUs were highest in the 12 °C and 21 °C treatments and decreased when exposed to drying-rewetting legacies or to two freezing-thawing cycles. In the fourth response group, OTUs were lower in samples with a history of one or two drying-rewetting cycles. In the fifth response group, OTUs were higher in samples with a history of two fluctuations (Fig. 5). At phylum level, abundance signatures of major responders seemed to involve either an increase or decrease after drying-rewetting for Proteobacteria, Actinobacteria and Acidobacteria. Firmicutes increased after one or two drying-rewetting cycles.

For 16S-DNA profiles, we identified three response groups involving 118 OTUs (*P* < 0.05; Fig. S12). In the first response group, OTUs were lowest in relative abundance after one or two drying-rewetting cycles. In the second response group, OTUs were highest after one or two drying-rewetting cycles. In the third response group, OTUs were also highest after one or two drying-rewetting cycles, but OTUs with the highest relative abundance were observed in soil with two drying-rewetting cycles. Proteobacteria decreased after one or more drying-rewetting cycles. Actinobacteria, Crenarchaeota, and Firmicutes increased after one or more drying-rewetting cycles.

### Response groups within ITS2

**Fig. 6:**
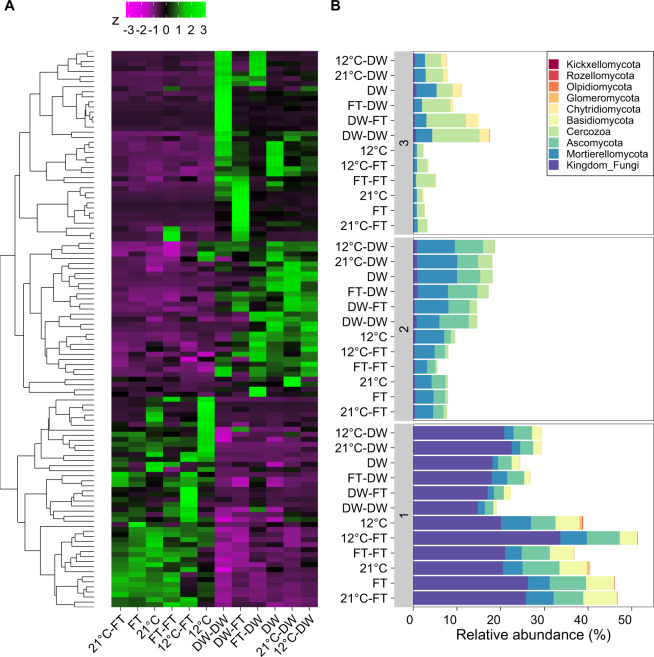
Response groups of ITS amplicons at cDNA level. **A** Presents heatmap of the *z*-score per OTU per treatment compared to the mean relative abundance. **B** Presents Barplot with the relative abundance per phylum per response group. Response groups are labeled with numbers and correspond to the groups in (**A**), 1 is the bottom group, 3 is the top group, the rest is also shown in order.

Three response groups were identified for 111 ITS2-cDNA profiles (*P* < 0.05; Fig. 6). OTUs belonging to response group one had lower relative abundance in soil with a history of one or two drying-rewetting cycles. In contrast, OTUs belonging to response group two had higher relative abundance in soil with a history of one or two drying-rewetting cycles. OTUs belonging to response group three showed different response patterns depending on legacies: OTUs were highest in soil subjected to two drying-rewetting cycles or a drying-rewetting cycle before a freezing-thawing cycle; were intermediate in soil with one drying-rewetting cycles; and were lowest in the soil without a history of fluctuations. OTUs belonging to Mortierellomycota and Ascomycota both increased and decreased in soil with a history of drying-rewetting compared to soil without it. OTUs belonging to the Basidiomycota and Glomeromycota decreased after one or two drying-rewetting cycles. OTUs belonging to the protists Cercozoa increased in after one or two drying-rewetting cycles. OTUs belonging to Chytridiomycota were highest in soil with a legacy of drying-rewetting. OTUs belonging to Rozellomycota and Kickxellomycota were lowest in soil with a legacy of drying-rewetting.

For ITS2-DNA profiles, we identified four response groups of 149 OTUs (*P* < 0.05; Fig. S13). The OTUs that belonged in response group one were highest in the soil subjected to two drying-rewetting cycles or one drying-rewetting cycle before one freezing-thawing cycle. In response group two, OTUs were higher in soil with a history of one or two drying-rewetting cycles. In response group three, OTUs were higher in soils exposed to one or two drying-rewetting or freezing-thawing cycles. In response group four, OTUs were lowest in soil with a history of one or two drying-rewetting cycles. Similar responses of OTUs were observed for both DNA and cDNA levels.

### Correlations between microbial relative abundance and CO_2_ emissions

**Fig. 7:**
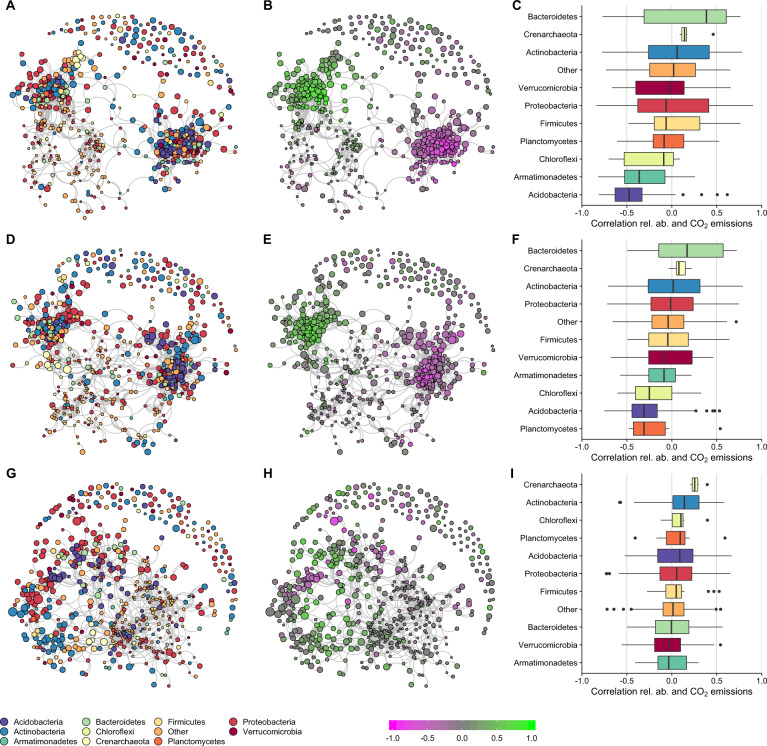
Co-response networks of the 16S OTUs at cDNA level and correlation between relative abundance and CO_2_ emissions. **A**, **B**, **C** Show Experiment 1, single treatments. **D**, **E**, **F** Show Experiment 2, single fluctuation followed by a drying-rewetting fluctuation. **G**, **H**, **I**, Show Experiment 3, single fluctuations followed by a freezing-thawing fluctuation. **A**, **D**, **G** Present co-response network with phylum overlay, legend below (**G**). Node sizes are indicative for the mean relative abundance. **B**, **E**, **H** Show the co-response network with correlation between relative abundance and CO_2_ emissions, legend is shown below (**H**). **C**, **F**, **I** Show boxplots with the correlations per OTU in selected phyla.

**Fig. 8:**
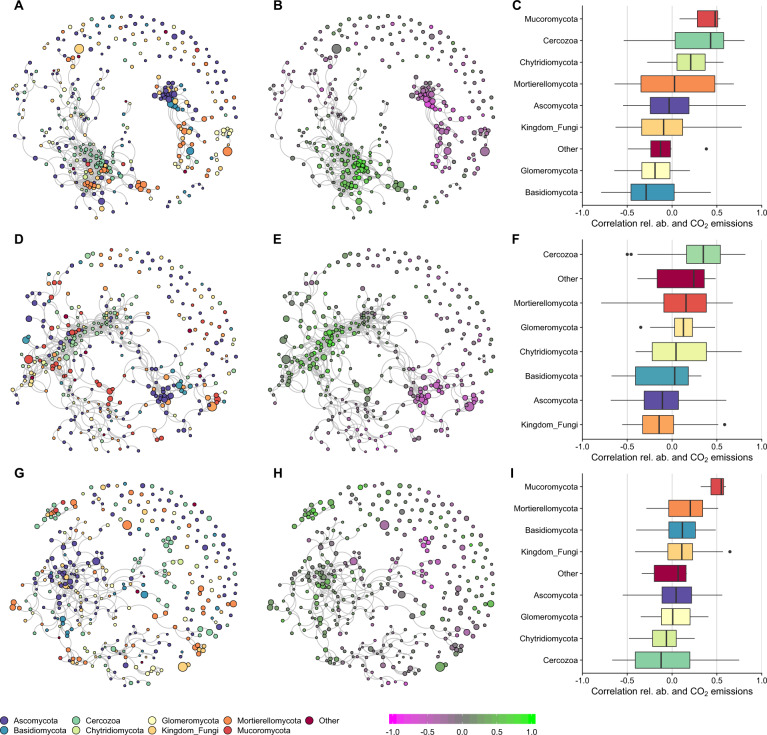
Co-response networks of the ITS OTUs at cDNA level and correlation between relative abundance and CO_2_ emissions. **A**, **B**, **C** Show Experiment 1, single fluctuations and controls. **D**, **E**, **F** Show Experiment 2, single fluctuations followed by a drying-rewetting fluctuation. **G**, **H**, **I** Show Experiment 3, single fluctuations followed by a freezing-thawing fluctuation. **A**, **D**, **G** Co-response network with phylum overlay, legend below (**G**). Node sizes are indicative for the mean relative abundance. **B**, **E**, **H** Show the co-response network with correlation between relative abundance and CO_2_ emissions, legend is shown below (**H**). **C**, **F**, **I** Show boxplots with the correlations per OTU in selected phyla.

Two large co-response clusters were found after a single fluctuation and after a follow-up drying-rewetting fluctuation for 16S-cDNA (Fig. 7) and ITS2-cDNA (Fig. 8) OTUs. For 16S-cDNA, Acidobacteria were mostly in the cluster negatively affected by drying-rewetting and were also negatively correlated with the release of CO_2_ upon rewetting. Actinobacteria and Proteobacteria were mostly in the cluster positively affected by drying-rewetting and were positively correlated with the release of CO_2_ upon rewetting (Fig. 7A-F). The formation of two main co-response clusters was mostly absent when the initial treatment was followed by a freezing-thawing cycle. For these treatments, lower correlation was observed between relative abundance and release of CO_2_ upon rewetting (Fig. 7G-I). For ITS-cDNA, Mortierellomycota and Cercozoa OTUs were mainly in the cluster negatively correlated with drying-rewetting and were positively correlated with the release of CO_2_ upon rewetting. Basidiomycota OTUs were mainly in the cluster positively correlated with drying-rewetting and were negatively correlated with the release of CO_2_ upon rewetting. The co-response clusters found after drying-rewetting and the positive and negative correlations with the release of CO_2_ were mostly absent after an additional freeze-thawing fluctuation (Fig. 8G-I). A similar picture was observed for correlations between CO_2_ and OTUs relative abundance for 16S-DNA and ITS2-DNA (Figs. S14, S15).

## Discussion

Our results did not support our hypothesis that drying-rewetting and freezing-thawing would lead to a microbiome that was more tolerant to an additional fluctuation. A history of a drying-rewetting, but not freezing-thawing, affected CO_2_ release and soil microbiome composition when the soil was exposed to an additional drying-rewetting or freezing-thawing cycle. Further, the microbiome composition, after one or two drying-rewetting cycles, became more similar to each other compared to the community composition in all other treatments without a drying-rewetting fluctuation (Figs. 5, 6). This finding supports prior work reporting that prokaryotes in soil subjected to experimental drying are more tolerant to an additional drying-rewetting cycle [21]. As such, changes in microbiome composition after one drying-rewetting cycle [e.g., 9, 16, 17] remain present and may affect individual microbial responses and overall community composition to an additional, potentially new fluctuation.

One explanation why communities were more robust to freezing-thawing than drying-rewetting fluctuations may be that drying-rewetting cycles are more physiologically stressful for soil microorganisms. A harsher environmental treatment may lead to more damage of drought-sensitive microorganisms [35, 36], and thus a reduction in microbial survival [13]. As such, a larger fraction of the surviving microbial community may enter into a dormant state [42, 43], potentially increasing the relative abundance of organisms like Firmicutes (Fig. 5). The soil was dried to ca. 1.3% wet weight, which is in line with other studies [9, 13, 23]. However, our experimental drying may have a stronger effect on water activity than freezing at −4 °C, which mimics the natural freezing temperatures soil microorganisms experience in a temperate ecosystem. In contrast, other studies reporting strong effects of freezing, use much lower freezing temperature, such as −15 °C or −20 °C [e.g., 33, 70]. A lower freezing temperature would decrease microbial activity more, as less water is available thus making more physiologically stressful conditions [71]. A lower freezing temperature also affects the microbial response upon thawing [7]. Alternatively, our freezing-thawing cycle may resulted in a more gradual change in water activity than the more abrupt shifts encountered during rewetting of dry soils, giving soil microorganisms more time to adapt to the altered osmotic pressure [5].

Our results support previous observations that multiple microbial phyla, with different traits, respond to a drying-rewetting cycle [16]. We clustered OTUs into distinct response groups, regardless of their phylogenetic origin. For example, OTUs belonging to Proteobacteria responded opportunistically or sensitively to one or more drying-rewetting cycle. This is in line with previous research, as Proteobacteria can be sensitive [32], tolerant [9], opportunistic [18] or have a mixed response strategy [17] to drying-rewetting cycles. Actinobacteria can also be tolerant [10], sensitive [9, 17], opportunistic [32] or have mixed response strategies [our study, 21] to drying-rewetting cycles. Phyla with a copiothrophic strategy may take advantage of resource availability following stress and are thus important for microbial succession into empty habitats left by less resilient microorganisms [16]. In addition, oligo- and copiothrophy are spread among phylogenetic groups [72] and so is the response of microorganisms to perturbations.

Our results did not support our hypothesis that fungi are more sensitive to freezing-thawing cycles than to drying-rewetting cycles. Fungal OTUs were more responsive to a drying-rewetting cycle than a freezing-thawing cycle. In addition, shifts in the fungal community mediated by one drying-rewetting cycle remained generally stable even after a second drying-rewetting or freezing-thawing cycle. Fungal communities can be less sensitive to drying-rewetting than bacterial communities [15, 17, 73], yet fungal community compositional shifts following drying-rewetting cycles can lead to lasting community changes [74]. Moreover, fungal communities may be more tolerant to freezing-thawing cycles than observed in previous studies in Artic regions [33, 51, 53, 54]. Other studies have used lower freezing temperatures that we did and these lower temperatures may increase fungal stress.

The group of protozoa picked up by our primers suggests an opportunistic strategy to drying-rewetting cycles for Cercozoa that may be important for changes in food-web dynamics upon recovery. The primers used to target the ITS2 region (gITS7-ITS4) also picked up non-fungal taxa, which is in line with previous observations [75]. Cercozoa increased in relative abundance following one or two drying-rewetting cycles. Cercozoa are generally abundant in soils and are important for soil food-web dynamics [76]. Protists live in the aquatic phase of soils [77] and are drought sensitive [78, 79]. Yet, Cercozoan species can be resilient to drought [80], as they can enter cystic forms that may survive unfavorable conditions [81, 82]. Upon recovery from drought, protists respond to wetter conditions more slowly than their prey, bacteria [79]. As Cercozoa correlated positively with CO_2_ emissions when soil had been exposed to drying-rewetting fluctuations (Fig. 8), food-web dynamics upon recovery may have created a cycle of predator-prey oscillations over time when sporulated protozoa may have the opportunity to feed upon fast-responding bacteria [8].

Similar to previous studies, the potentially active, RNA-based, fraction of the prokaryote OTUs responded differently to the drying-rewetting fluctuations than the total (active + inactive pool) DNA-based fraction [9, 15]. Our results suggest that the presence of an OTU at the DNA level may not indicate that the observed species is active. For example, we observed that DNA from Thaumarchaea responded opportunistically in soils with a history of drying-rewetting (Fig. S13), which is in line with earlier work [16, 83, 84]. However, this pattern disappeared when observing the RNA response (Fig. 5, Crenarchaeota belong to Thaumarchaea). Thus, our study suggests that Thaumarchaea total abundance may increase, but their potential activity remains the same. This confirms that high levels of DNA in soil do not indicate that the prokaryote is in an active state [85, 86].

Our results may indicate that a shift toward a more drought tolerant microbiome may reduce respiration upon a second drying-rewetting cycle. The CO_2_ flux upon rewetting was lower when soil experienced a previous drying-rewetting cycle compared to soil without this history. This result reflects prior work where CO_2_ flux declined following multiple drying-rewetting cycles [26, 27, 87]. One mechanism for a decreased respiration upon multiple drying-rewetting cycles may be a decrease in the supply rates of organic matter for microbial respiration due to the high CO_2_ pulse during the first drying-rewetting fluctuation [22, 88]. However, an altered microbiome composition after one drying-rewetting cycle [16, 17] may affect the composition present upon the second drying-rewetting cycle (this study). Moreover, the changed microbiome composition seems to affect the correlation with CO_2_ emissions (Figs. 7, 8). The correlations between CO_2_ emissions and microbial community are based on relative abundance data, which does not give information on the total abundance of changes in the microbial community [89]. As total microbial community size seems to be unaffected by our treatments (Figs. S4, S5) and microbial community shifts are also present in sequence read counts (Figs. 3, 4), our results indicate a shift in microbial community composition. These changed microbiomes may not only affect carbon cycling, but also affect other ecosystem functions. For example, drought-exposed microbiomes affect individual and community responses of plant species to additional perturbations [90, 91].

The positive correlations between OTUs and CO_2_ emissions generally occurs within phyla Actinobacteria and Proteobacteria for prokaryotes and within phyla Cercozoa and Mortierellomycota for ITS2. These correlations may be caused by responses at a finer taxonomic resolution. For example, some Actinobacteria, like *Arthrobacter spp*., increase after one or two drying-rewetting fluctuations. This genus may tolerate drought, as it can transit between a vegetative and dormant state [92]. High CO_2_ emission may be released upon rewetting via germinating spores [93]. Other Actinobacteria, like *Thermomonospraceae*, responded sensitive to drought and may contribute less to CO_2_ emissions post-disturbance. There are many Proteobacteria OTUs that represent copiotrophs. These may contribute via an opportunistic strategy to the CO_2_ flux upon rewetting if they tolerate drought. As soil communities are complex, it is likely that numerous community members not included in our study responded to the fluctuations. For example, soil viruses are often linked to soil hydration and microbial community dynamics [94], thus exploring viral responses to drying-rewetting cycles is an exciting avenue for future work.

In conclusion, a history of drying-rewetting affected the soil microbiome composition and its responses to future fluctuations. Soil microorganisms that have experienced previous drying-rewetting cycles may impact the community response to future perturbations (e.g., [95, 96]). In addition, changes in soil microbiomes following drought and freezing may affect soil processes and ecosystem services in the future. For example, drought-exposed microbiomes affect the carbon cycle (this study) and the response of plant species to additional perturbations [90, 91]. As such, microbiome changes due to a drying-rewetting history may have implications for functioning in ecosystems that experience increasing frequency and/or intensity of weather perturbations due to global change.

## Supplementary information

Supplemental Material
