# Supplementary material for: Soil microbial legacies differ following drying-rewetting and freezing-thawing cycles
Source: ISME J. 2021 Jan 6;15(4):1207–21. doi: 10.1038/s41396-020-00844-3 (PMC8115648; doi:10.1038/s41396-020-00844-3)
Supplement: Supplementary file 1 — Supplemental Material [file 41396_2020_844_MOESM1_ESM.pdf]

Additional methods

qPCR of prokaryotes and fungi

Bacterial abundance in the soil samples was measured by quantifying the V3 region of 16S rDNA as described previously [1]. Briefly, the PCR mixture contained 1 µl of the forward EUB338 and 1 µl of the reverse EUB518 primer [2] (0.2 µM final concentration of both primers), 12.5 µl of the Brilliant III Ultra-Fast SYBR® Green QPCR Master Mix (Agilent), 8.5 µl of molecular grade water and 2 µl of 10x diluted cDNA or 100x diluted DNA template. The total amount of DNA added to the qPCR reaction was around 1 ng and the total amount of cDNA added was in the range of 3-20 ng. PCR was performed as follows: 95°C for 10 min, followed by 45 cycles of denaturation at 95°C for 10s, annealing at 56°C for 10s, and extension at 72°C for 13s and a final dissociation curve. The standard curve was made from DNA isolated from *E.coli* [3]. Fungal abundance in the soil samples was measured by quantifying the ITS2 region as described previously [4]. The PCR mixture contained 1 µl of the forward primer gITS7 [5], 1 µl of the reverse primer ITS4 [6] (0.2 µM final concentration of both primers), 10 µl of Brilliant III Ultra-Fast SYBR® Green QPCR Master Mix (Agilent), 7 µl of RNA free water and 1 µl of 10x diluted cDNA or 100x diluted DNA template. PCR conditions were 95 °C for 2 min, followed by 40 cycles of 95 °C for 10 s, 56 °C for 20 s, 72 °C for 45 s, an extension step of 72 °C for 6 min and a final dissociation curve. The standard curve was made from a plasmid that contained the ITS fragment of *Aureobasidium pullulans*.

Preparation for sequencing of prokaryotic and fungal communities

The sequencing to determine the composition of prokaryotic and fungal communities for the 16S rRNA and ITS2 gene and transcripts were done as described by Borg Dahl et al. [7]. For 16S, we used the 314F and 806R primers [8-10]. For ITS2 region, we used the primers gITS7 [5] and ITS4 [6]. The PCR amplification was done in two steps. The first PCR mixture contained 15.75 µl molecular grade water, 5 µl PCRBIO 5 x reaction buffer, 0.25 µl PCRBIO Hifi Polymerase (PCR Biosystems, London, UK), 1 µl of each forward and reverse primer (0.2 µM final concentration) and 2 µl of diluted template (see above). The PCR conditions were the following: 95 °C for 1 min followed by 30 cycles of 95 °C for 15 seconds, 56°C for 15 seconds and 72°C for 30 seconds. Then there was a final extension at 70 °C for 3 min. PCR products were checked on agarose gels. First PCR was performed in technical triplicates to minimize PCR bias [11], which were pooled after the first PCR. After pooling technical replicates, PCR products were purified by Agencourt AMPure XP (Beckman Coulter). Addition of adaptors and indices was done in a second PCR reaction that contained 15.75 µl molecular grade water, 5 µl PCRBIO 5 x reaction buffer, 0.25 µl PCRBIO Hifi Polymerase (PCR biosystems), 1 µl of each of the forward and reverse primer that contained adaptor and index and 2 µl of the cleaned PCR product (see above). The PCR conditions were the following: 95 °C for 1 min followed by 15 cycles of 95 °C for 15 seconds, 56°C for 15 seconds and 72°C for 30 seconds. Then there was a final extension at 70 °C for 3 min. The PCR product were checked on a gel and purified using Agencourt AMPure XP (Beckman Coulter).

The cleaned and indexed PCR products were pooled by adding equal amounts of DNA to a 1.5 ml tube. The sample pool was concentrated using the DNA clean-and-concentrator-5 kit (Zymo Research, Irvine, California, USA) and prepared for sequencing using the MiSeq reagent kit v2 (500 cycles) and paired-end sequenced on the Illumina MiSeq platform (Illumina Inc., San Diego, California, USA) at the Section of Microbiology, University of Copenhagen.

Annotation

For 16S cDNA and DNA, raw reads were trimmed of adaptor, indices and primer sequences using *cutadapt* [12]. Only read pairs having both primers were retained. Trimmed sequences were merged (*assemble_pairs*) and clustered in OTUs (*cluster_otus*) with *UPARSE-OTU* algorithm [13] (97% sequence similarity, custom *BioDSL* script, https://github.com/maasha/BioDSL). Taxonomic annotation of OTUs was performed with *mothur* [14] naïve Bayes' classifier using Ribosomal Database Project database (*RDP*, trainset 16 [15], https://www.mothur.org/wiki/RDP_reference_files). This implementation has been described [16] OTUs affiliated to mitochondria, chloroplasts, Eukaryotes or not affiliated to a Domain were removed (0.33% of total OTUs).

For ITS2 region cDNA and DNA, raw sequence reads were treated as above. Trimmed sequences were processed using *PIPITS* workflow [17]. Primer-free sequences were merged using PEAR [18] and quality-filtered using *FASTX-Toolkit* (Hannon 2010). ITS2 sequences were extracted using *ITSx* [19] and clustered into OTUs using *VSEARCH* and a (97% sequence similarity). Representative OTU sequences were chimera-checked using *UNITE-UCHIME* reference dataset. Taxonomy of chimera-free OTUs was assigned with *RDP* classifier [16] against the *UNITE* fungal ITS database version 7.2 [20]. OTUs without Domain affiliation were removed (2.73% of total OTUs).

References

1. Nunes I, Jurburg S, Jacquiod S, Brejnrod A, Falcão Salles J, Priemé A, et al. Soil bacteria show different tolerance ranges to an unprecedented disturbance. Biol Fertility Soils. 2018; 54 (2): 189-202.
2. Fierer N, Jackson JA, Vilgalys R, Jackson RB. Assessment of soil microbial community structure by use of taxon-specific quantitative pcr assays. Appl Environ Microbiol. 2005; 71 (7): 4117-20.
3. Schostag M, Stibal M, Jacobsen CS, Bælum J, Taş N, Elberling B, et al. Distinct summer and winter bacterial communities in the active layer of svalbard permafrost revealed by DNA- and rna-based analyses. Front Microbiol. 2015; 6: 399.
4. Christiansen CT, Haugwitz MS, Priemé A, Nielsen CS, Elberling B, Michelsen A, et al. Enhanced summer warming reduces fungal decomposer diversity and litter mass loss more strongly in dry than in wet tundra. Global Change Biol. 2017; 23 (1): 406-20.
5. Ihrmark K, Bödeker ITM, Cruz-Martinez K, Friberg H, Kubartova A, Schenck J, et al. New primers to amplify the fungal its2 region – evaluation by 454-sequencing of artificial and natural communities. FEMS Microbiol Ecol. 2012; 82 (3): 666-77.
6. White TJ, Bruns T, Lee S, Taylor J. 38 - amplification and direct sequencing of fungal ribosomal rna genes for phylogenetics. Pcr protocols. San Diego: Academic Press; 1990. p. 315-22.
7. Borg Dahl M, Brejnrod AD, Russel J, Sørensen SJ, Schnittler M. Different degrees of niche differentiation for bacteria, fungi, and myxomycetes within an elevational transect in the german alps. Microb Ecol. 2019.
8. Sundberg C, Al-Soud WA, Larsson M, Alm E, Yekta SS, Svensson BH, et al. 454 pyrosequencing analyses of bacterial and archaeal richness in 21 full-scale biogas digesters. FEMS Microbiol Ecol. 2013; 85 (3): 612-26.
9. Yu Y, Lee C, Kim J, Hwang S. Group-specific primer and probe sets to detect methanogenic communities using quantitative real-time polymerase chain reaction. Biotechnol Bioeng. 2005; 89 (6): 670-9.

10. Neefs JM, Van de Peer Y, Hendriks L, De Wachter R. Compilation of small ribosomal subunit rna sequences. Nucleic Acids Res. 1990; 18 Suppl (Suppl): 2237-317.
11. Schöler A, Jacquiod S, Vestergaard G, Schulz S, Schloter M. Analysis of soil microbial communities based on amplicon sequencing of marker genes. Biol Fertility Soils. 2017; 53 (5): 485-9.
12. Martin M. Cutadapt removes adapter sequences from high-throughput sequencing reads. EMBnetjournal. 2011; 17 (1): 3.
13. Edgar RC. Uparse: Highly accurate otu sequences from microbial amplicon reads. Nat Methods. 2013; 10: 996.
14. Schloss PD, Westcott SL, Ryabin T, Hall JR, Hartmann M, Hollister EB, et al. Introducing mothur: Open-source, platform-independent, community-supported software for describing and comparing microbial communities. Appl Environ Microbiol. 2009; 75 (23): 7537-41.
15. Cole JR, Wang Q, Fish JA, Chai B, McGarrell DM, Sun Y, et al. Ribosomal database project: Data and tools for high throughput rrna analysis. Nucleic Acids Res. 2013; 42 (D1): D633-D42.
16. Wang Q, Garrity GM, Tiedje JM, Cole JR. Naïve bayesian classifier for rapid assignment of rrna sequences into the new bacterial taxonomy. Appl Environ Microbiol. 2007; 73 (16): 5261-7.
17. Gweon HS, Oliver A, Taylor J, Booth T, Gibbs M, Read DS, et al. Pipits: An automated pipeline for analyses of fungal internal transcribed spacer sequences from the illumina sequencing platform. Methods in Ecology and Evolution. 2015; 6 (8): 973-80.
18. Zhang J, Kobert K, Flouri T, Stamatakis A. Pear: A fast and accurate illumina paired-end read merger. Bioinformatics (Oxford, England). 2014; 30 (5): 614-20.
19. Bengtsson-Palme J, Ryberg M, Hartmann M, Branco S, Wang Z, Godhe A, et al. Improved software detection and extraction of its1 and its2 from ribosomal its sequences of fungi and other eukaryotes for analysis of environmental sequencing data. Methods in Ecology and Evolution. 2013; 4 (10): 914-9.
20. UNITECommunity. Unite mothur release. 2017.

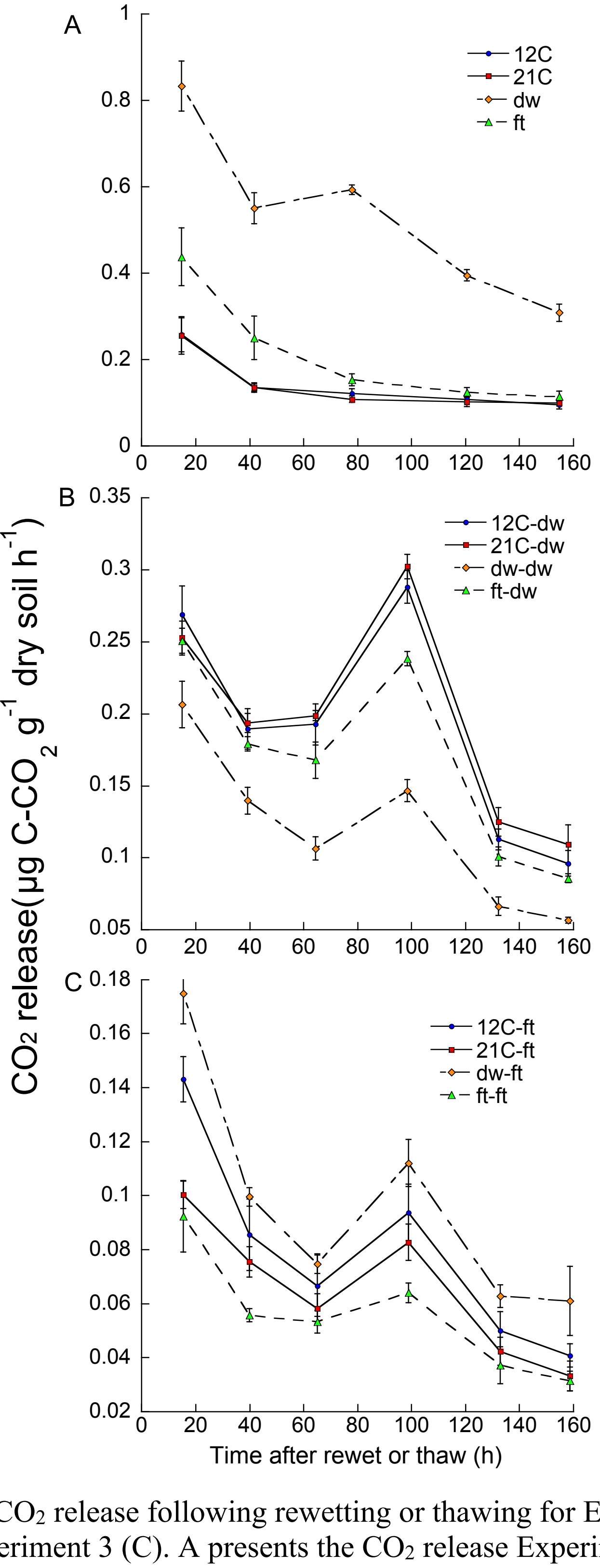

Fig. S1 CO_2 release following rewetting or thawing for Experiment 1 (A), Experiment 2 (B) and Experiment 3 (C). A presents the CO_2 release Experiment 1, which tested the effect of drying-rewetting versus freezing-thawing. B presents the CO_2 release in Experiment 2, which tested the legacy of freezing-thawing on drying-rewetting. C presents the CO_2 release in Experiment 3, which tested the legacy of drying-rewetting on freezing-thawing. 12ºC indicates pre-incubation at 12ºC, while 21ºC indicates pre-incubation at 21ºC. DW indicates drying-rewetting cycle. FT indicates freezing-thawing cycle.

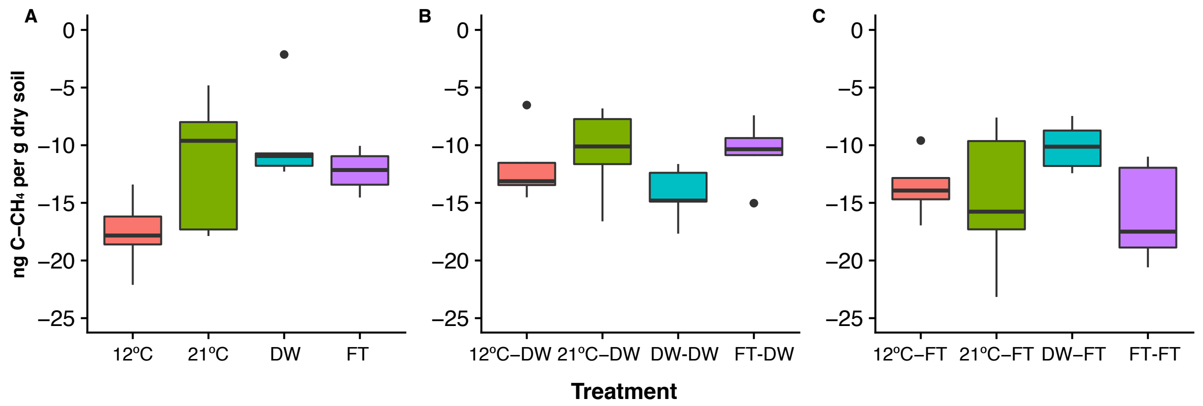

Fig. S2 CH_4 uptake at 12ºC by soil samples during a week upon rewetting and thawing. A presents the CH_4 uptake in Experiment 1, which tested the effect of drying-rewetting versus freezing-thawing. B presents the CH_4 uptake in Experiment 2, which tested the legacy of freezing-thawing on drying-rewetting. C presents the CH_4 uptake in Experiment 3, which tested the legacy of drying-rewetting on freezing-thawing. 12ºC indicates pre-incubation at 12ºC, while 21ºC indicates pre-incubation at 21ºC. DW indicates drying-rewetting cycle. FT indicates freezing-thawing cycle.

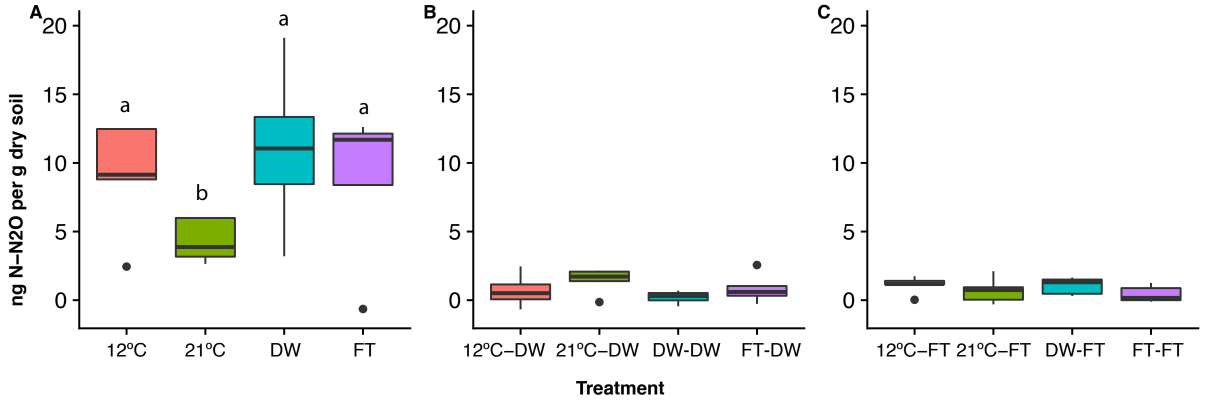

Fig. S3 N_2O released by soil samples incubated at 12ºC during a week upon rewetting and thawing. A presents the N_2O released in Experiment 1, which tested the effect of drying-rewetting versus freezing-thawing. B presents the N_2O released in Experiment 2, which tested the legacy of freezing-thawing on drying-rewetting. C presents the N_2O released in Experiment 3, which tested the legacy of drying-rewetting on freezing-thawing. 12ºC indicates pre-incubation at 12ºC, while 21ºC indicates pre-incubation at 21ºC. DW indicates drying-rewetting cycle. FT indicates freezing-thawing cycle. Different superscript letters denote significant different rates from the post hoc test at $P < 0.05$.

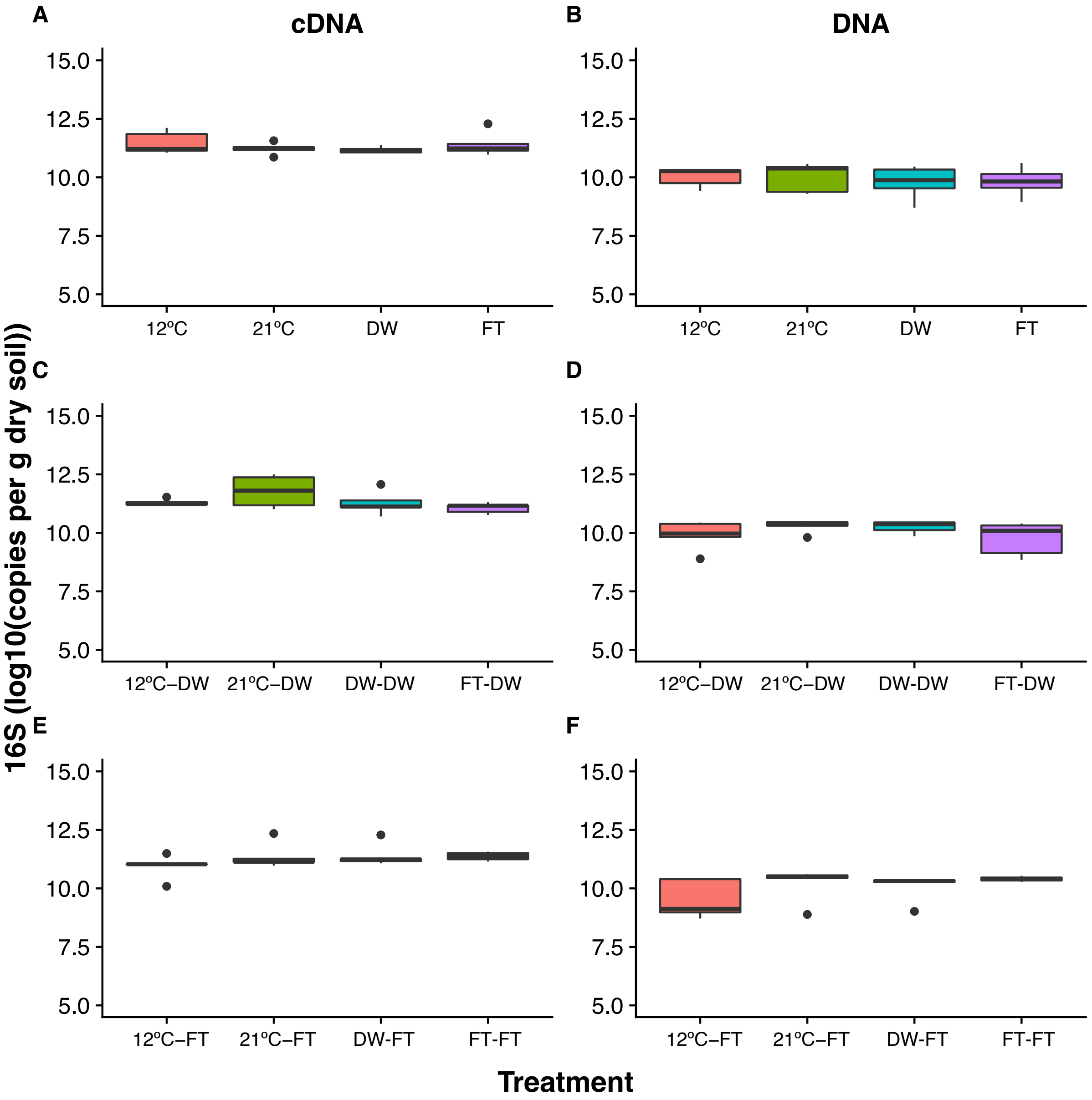

Fig S4 Abundance of 16S cDNA reads (A,C,E) and DNA reads (B,D,F) according to treatments as measured by qPCR. The treatments are described in fig. 1. A,B presents the abundance in Experiment 1, which tested the effect of drying-rewetting versus freezing-thawing. C, D presents the abundance in Experiment 2, which tested the legacy of freezing-thawing on drying-rewetting. E, F presents the abundance in Experiment 3, which tested the legacy of drying-rewetting on freezing-thawing. 12ºC indicates pre-incubation at 12ºC, while 21ºC indicates pre-incubation at 21ºC. DW indicates a drying-rewetting cycle. FT indicates a freezing-thawing cycle. Different superscript letters denote differences at $P < 0.05$ for a post hoc test for the mixed model.

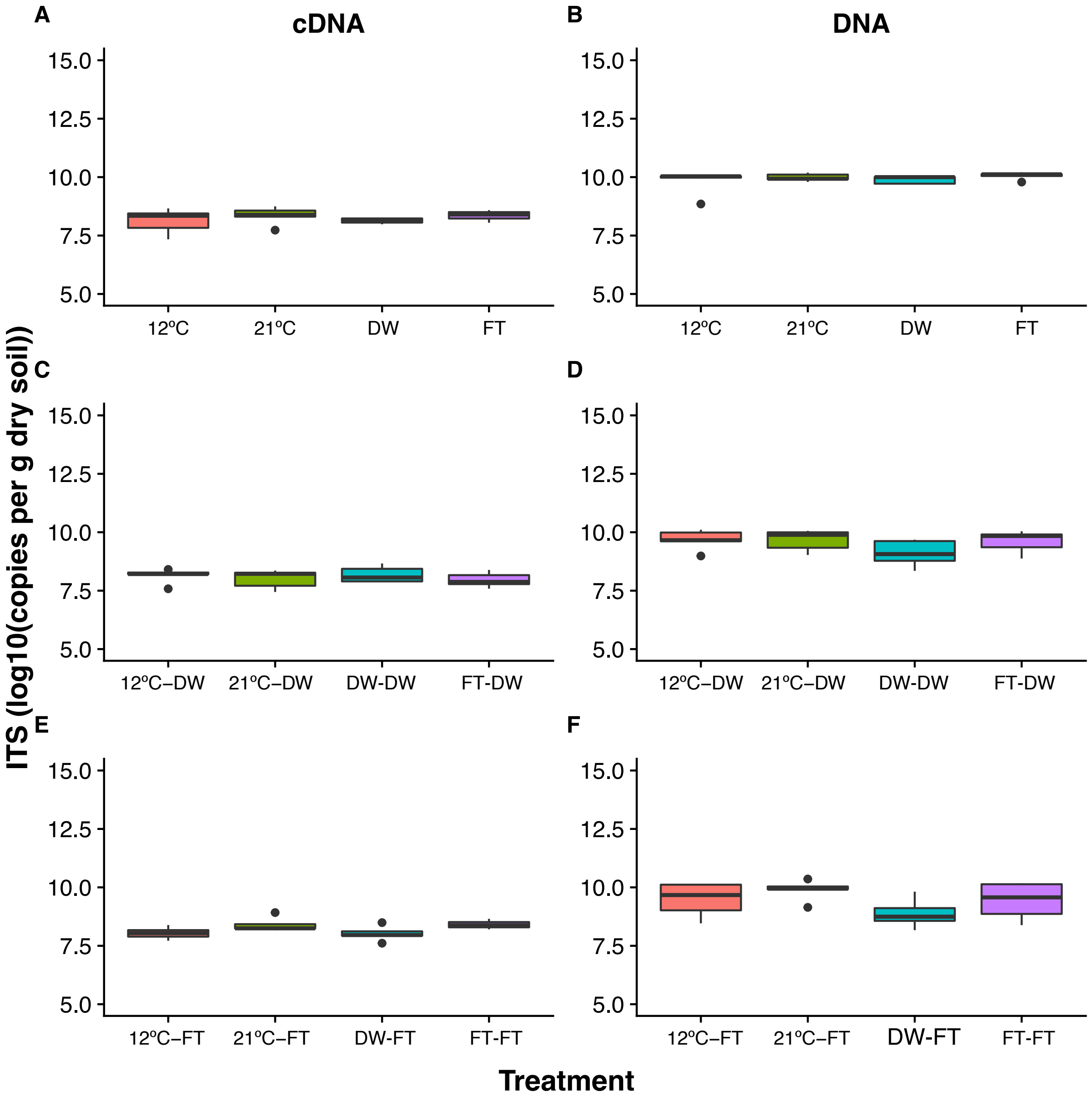

Fig. S5 Abundance of ITS cDNA reads (A,C,E) and DNA reads (B,D,F) according to treatments as measured by qPCR. The treatments are described in fig. 1. A,B presents the abundance in Experiment 1, which tested the effect of drying-rewetting versus freezing-thawing. C,D presents the abundance in Experiment 2, which tested the legacy of freezing-thawing on drying-rewetting. E,F presents the abundance in Experiment 3, which tested the legacy of drying-rewetting on freezing-thawing. 12ºC indicates pre-incubation at 12ºC, while 21ºC indicates pre-incubation at 21ºC. DW indicates a drying-rewetting cycle. FT indicates a freezing-thawing cycle. Different superscript letters denote differences at $P < 0.05$ for a post hoc test for the mixed model.

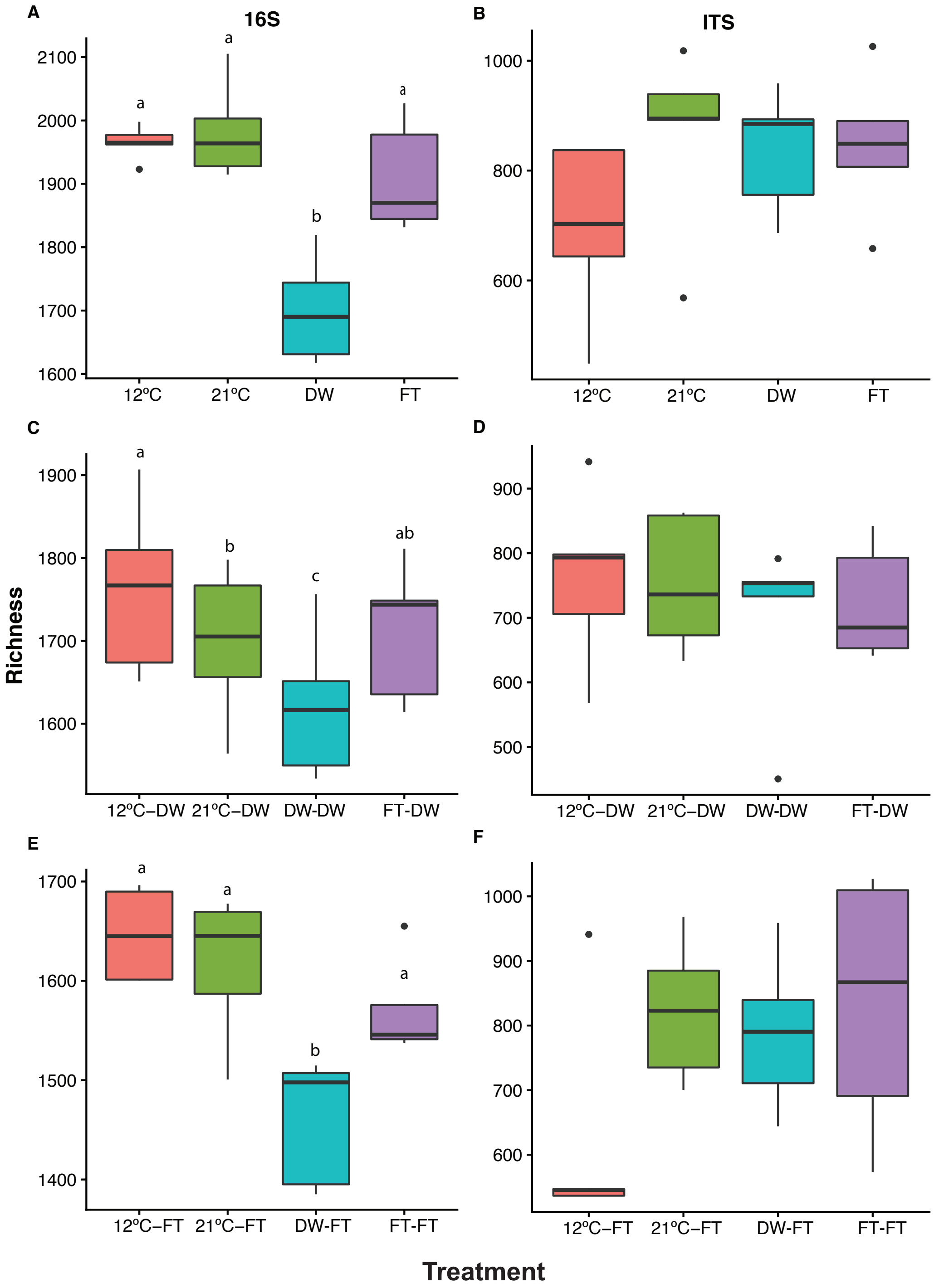

Fig. S6 Richness of 16S rRNA (A,C,E) and ITS amplicon profiles (B,D,F) for cDNA according to treatments. The treatments are described in figure 1. A,B presents the richness in Experiment 1, which tested the effect of drying-rewetting versus freezing-thawing. C,D presents the richness in Experiment 2, which tested the legacy of freezing-thawing on drying-rewetting. E,F presents the richness in Experiment 3, which tested the legacy of drying-rewetting on freezing-thawing. 12ºC indicates pre-incubation at 12ºC, while 21ºC indicates pre-incubation at 21ºC. DW indicates a drying-rewetting cycle. FT indicates a freezing-thawing cycle. Different superscript letters denote differences at $P < 0.05$ for a post hoc test for the mixed model.

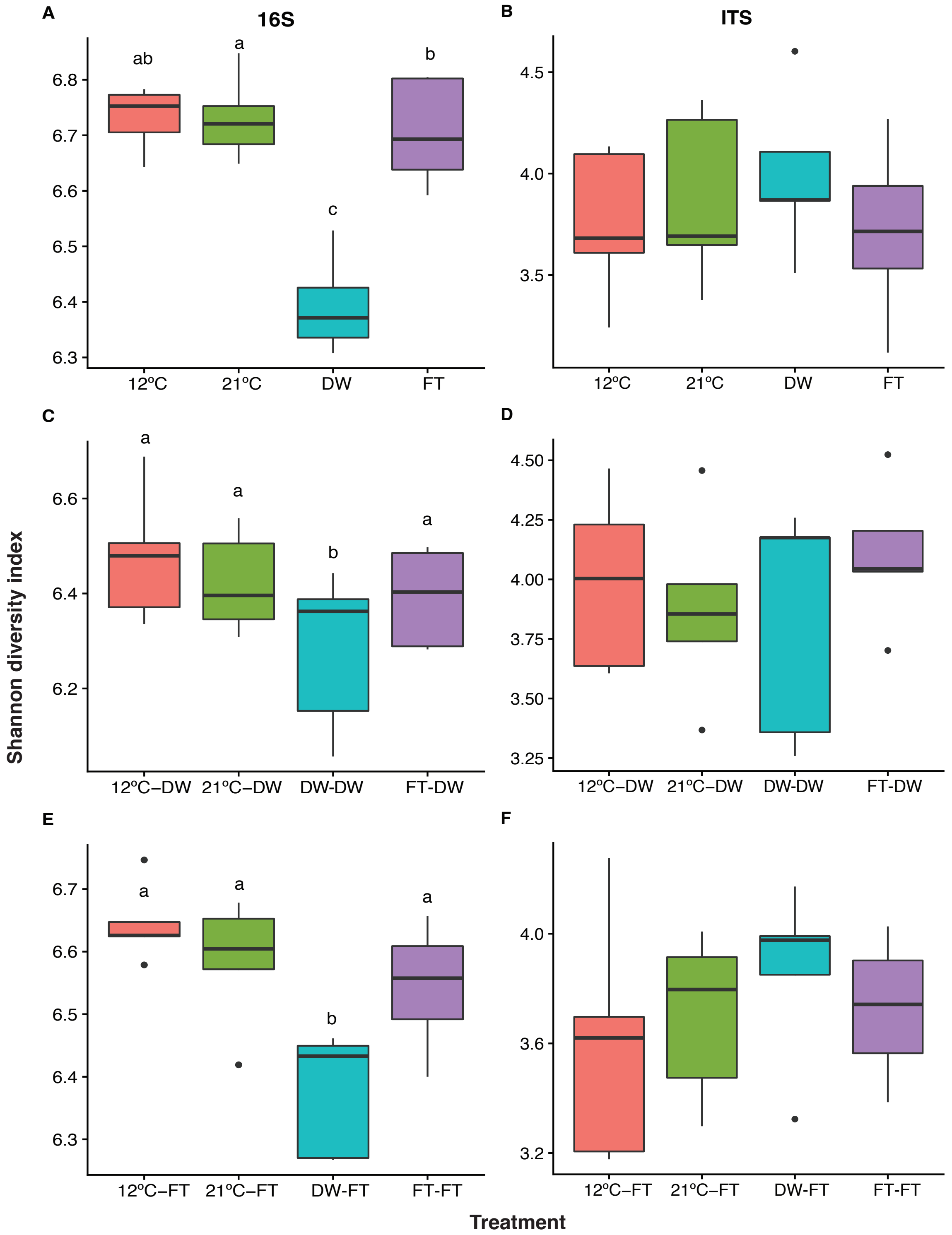

Fig. S7 Shannon-Weaver diversity index of 16S rRNA (A,C,E) and ITS amplicon profiles (B,D,F) for cDNA according to treatments. The treatments are described in figure 1. A,B presents the r Shannon diversity index in Experiment 1, which tested the effect of drying-rewetting versus freezing-thawing. C,D presents the Shannon diversity index in Experiment 2, which tested the legacy of freezing-thawing on drying-rewetting. E,F presents the Shannon diversity index in Experiment 3, which tested the legacy of drying-rewetting on freezing-thawing. 12ºC indicates pre-incubation at 12ºC, while 21ºC indicates pre-incubation at 21ºC. DW indicates a drying-rewetting cycle. FT indicates a freezing-thawing cycle. Different superscript letters denote differences at $P < 0.05$ for a post hoc test for the mixed model.

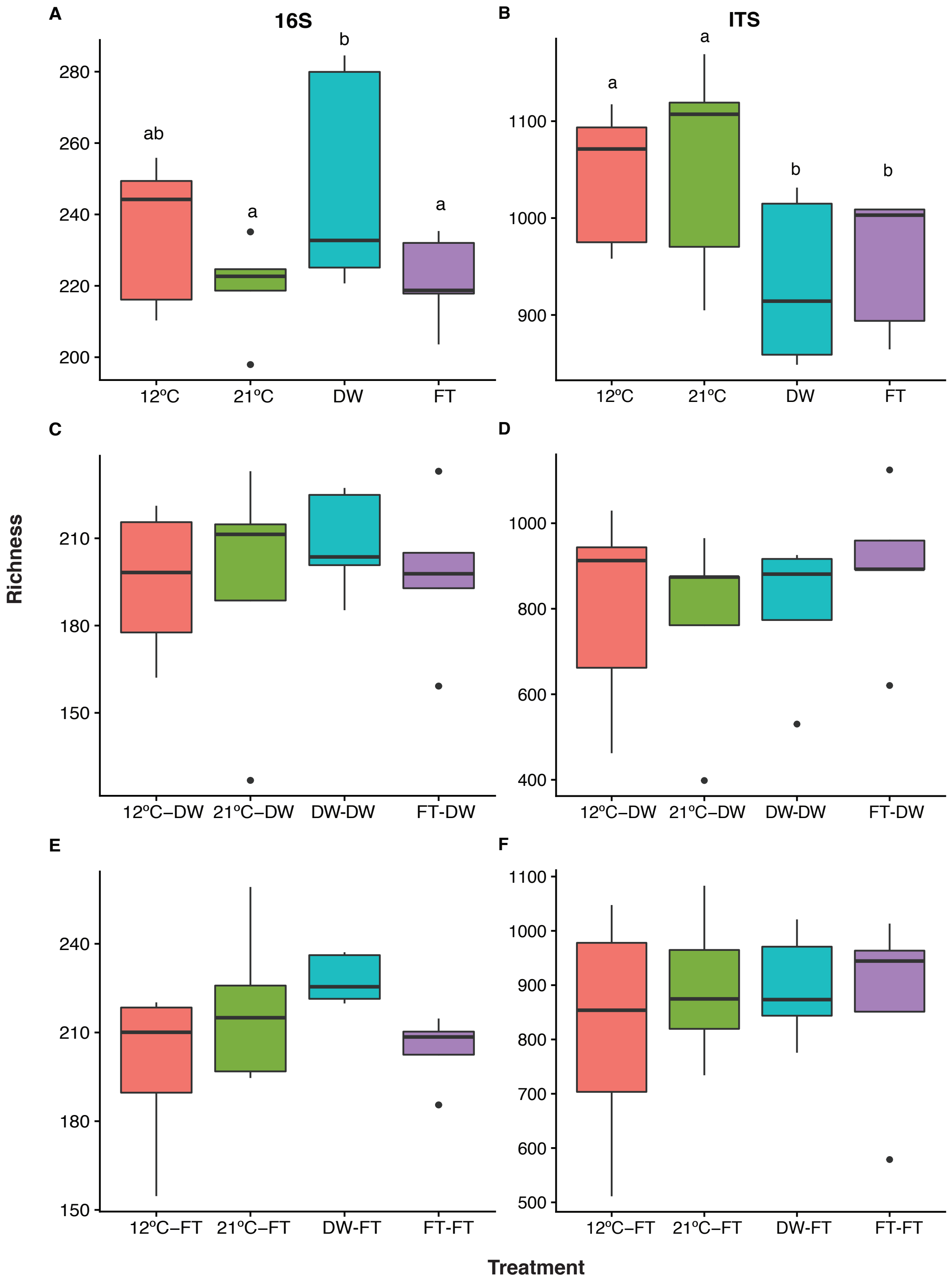

Fig. S8 Richness of 16S (A,C,E) and ITS amplicon profiles (B,D,F) for DNA according to treatments. The treatments are described in figure 1. A,B presents the richness in Experiment 1, which tested the effect of drying-rewetting versus freezing-thawing. C,D presents the richness in Experiment 2, which tested the legacy of freezing-thawing on drying-rewetting. E,F presents the richness in Experiment 3, which tested the legacy of drying-rewetting on freezing-thawing. 12ºC indicates pre-incubation at 12ºC, while 21ºC indicates pre-incubation at 21ºC. DW indicates a drying-rewetting cycle. FT indicates a freezing-thawing cycle. Different superscript letters denote differences at $P < 0.05$ for a post hoc test for the mixed model.

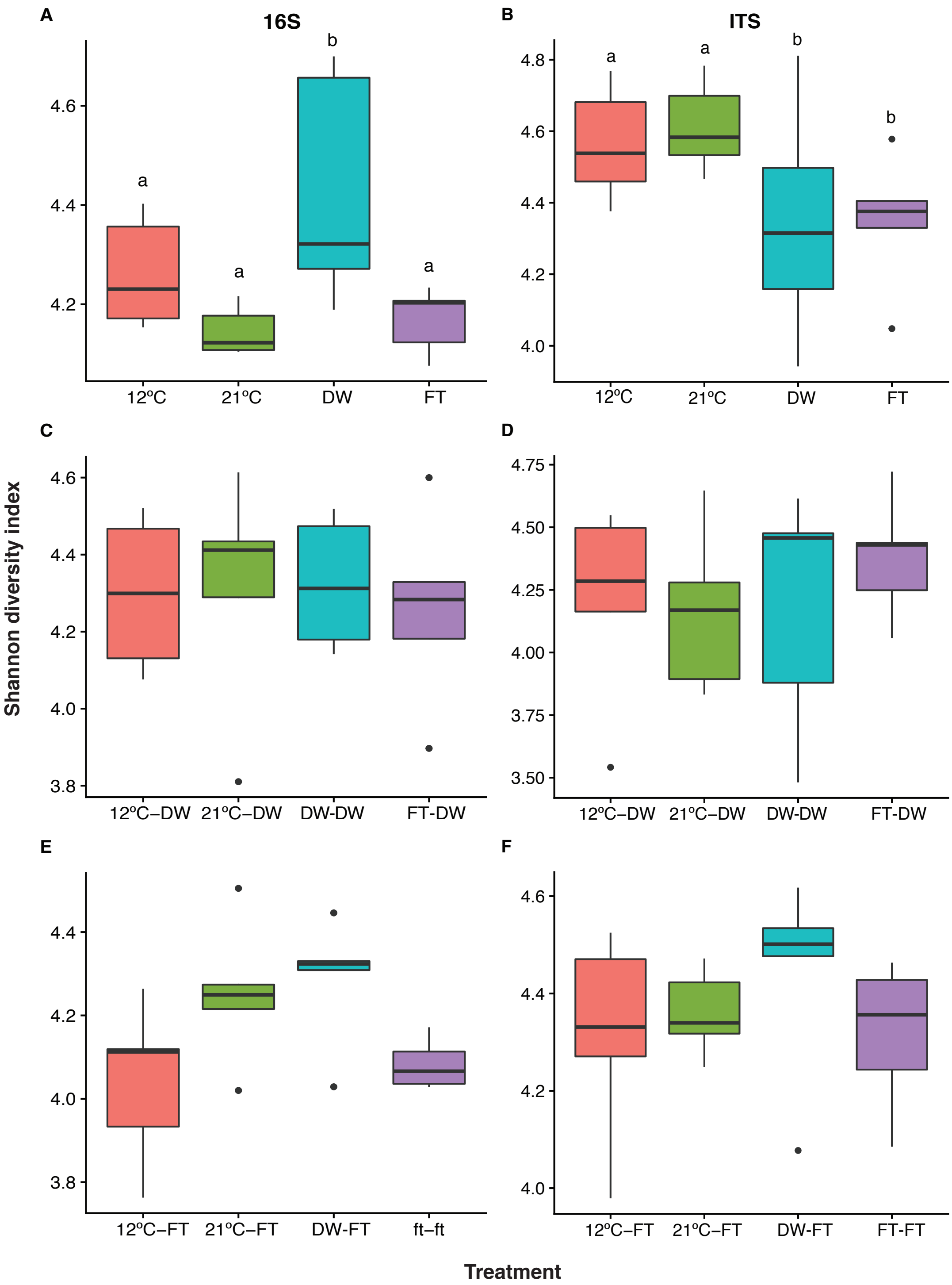

Fig. S9 Shannon diversity index of 16S (A,C,E) and ITS amplicon profiles for DNA (B,D,F) according to treatments. The treatments are described in figure 1. A,B presents the r Shannon diversity index in Experiment 1, which tested the effect of drying-rewetting versus freezing-thawing. C,D presents the Shannon diversity index in Experiment 2, which tested the legacy of freezing-thawing on drying-rewetting. E,F presents the Shannon diversity index in Experiment 3, which tested the legacy of drying-rewetting on freezing-thawing. 12ºC indicates pre-incubation at 12ºC, while 21ºC indicates pre-incubation at 21ºC. DW indicates a drying-rewetting cycle. FT indicates a freezing-thawing cycle. Different superscript letters denote differences at $P < 0.05$ for a post hoc test for the mixed model.

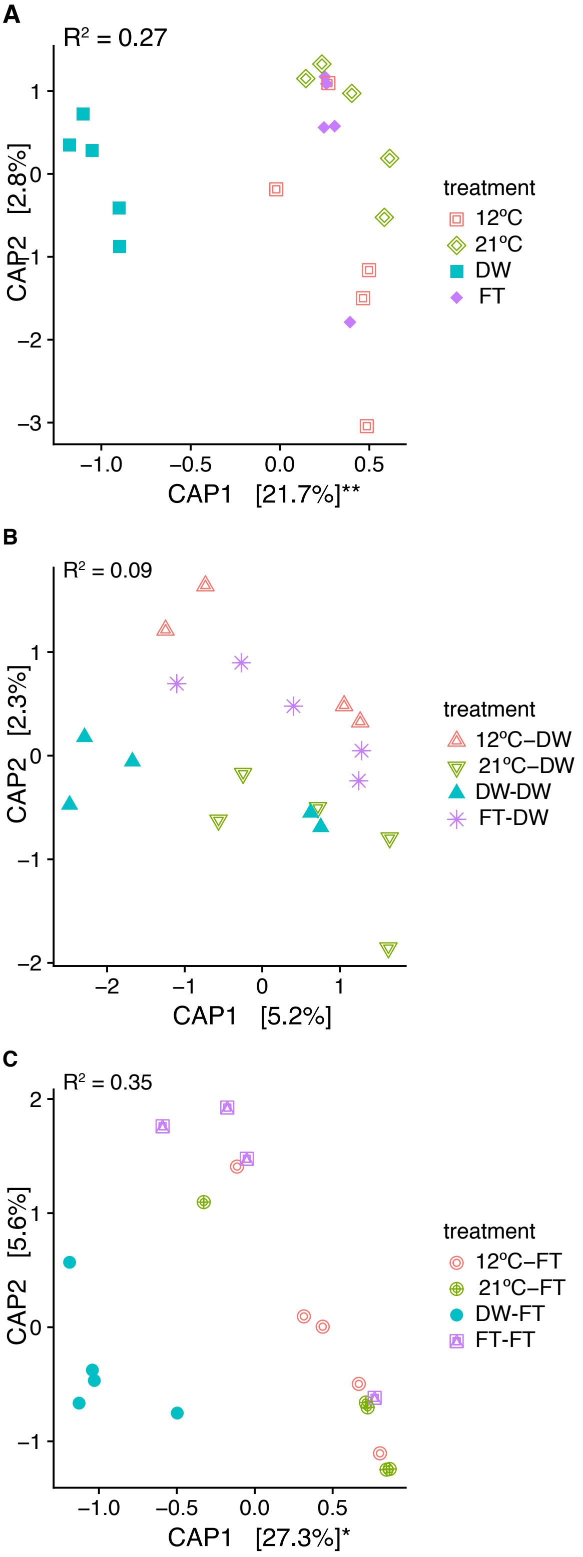

Fig. S10 Partial distance-based redundancy analysis of prokaryotes for DNA on Bray-Curtis dissimilarity using capscale ordination. A is Experiment 1 where we tested if a drying-rewetting (DW) or a freezing-thawing (FT) cycle leave different legacies in the prokaryote community. B is from Experiment 2 where we tested how the different legacies affected the microbial response to a drying-rewetting cycle. C is from Experiment 3 where we tested how different legacies affected the microbial communities after an additional FT cycle. 12ºC indicates pre-incubation at 12ºC, while 21ºC indicates pre-incubation at 21ºC (see Fig. 1). Significance of axes is tested with a permutation test by axis: ** P<0.01; ***p<0.001.

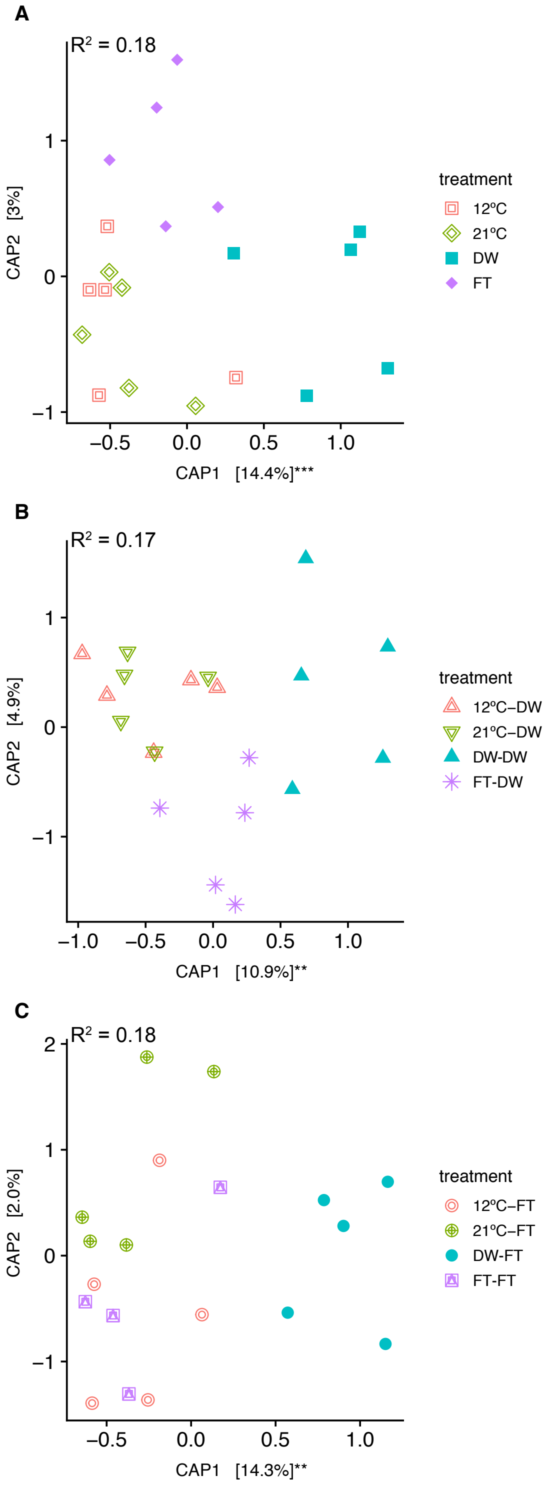

Fig. S11 Partial distance-based redundancy analysis of ITS for DNA on Bray-Curtis dissimilarity using capscale ordination. A is Experiment 1 where we tested if a drying-rewetting (DW) or a freezing-thawing (FT) cycle leave different legacies in the prokaryote community. B is from Experiment 2 where we tested how the different legacies affected the microbial response to a drying-rewetting cycle. C is from Experiment 3 where we tested how different legacies affected the microbial communities after an additional FT cycle. 12ºC indicates pre-incubation at 12ºC, while 21ºC indicates pre-incubation at 21ºC (see Fig. 1). Significance of axes is tested with a permutation test by axis: ** P<0.01; ***p<0.001.

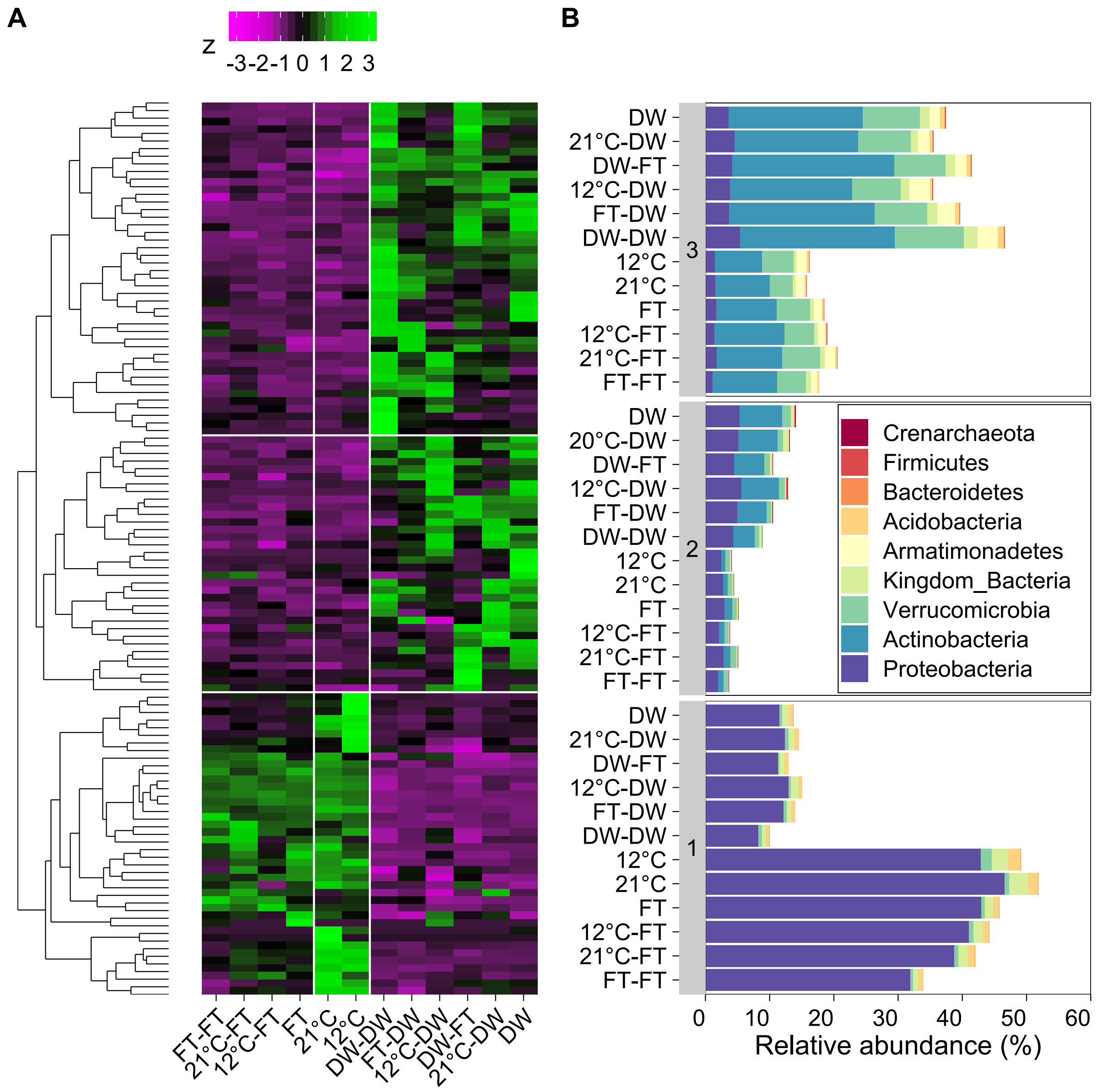

Fig. S12 Response groups of 16S amplicons at DNA level. **A**) Heatmap of the z-score per OTU per treatment compared to the mean relative abundance. **B**) Barplot with the relative abundance per phylum per response group. Response groups are labeled with numbers and correspond to the groups in A, 1 is the bottom group, 3 is the top group, the rest is also shown in order.

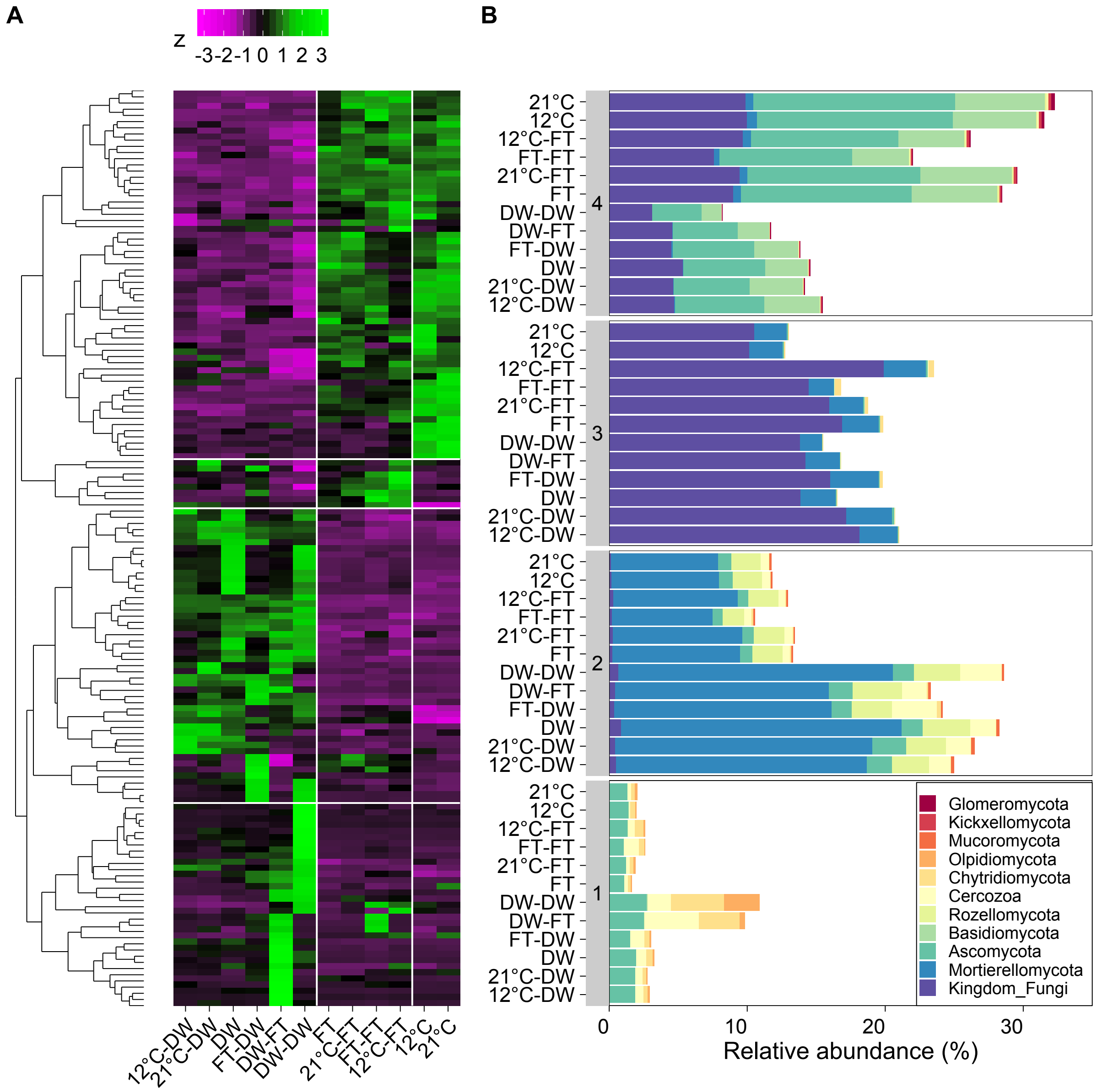

Fig. S13 Response groups of ITS amplicons at cDNA level. **A**) Heatmap of the z-score per OTU per treatment compared to the mean relative abundance. **B**) Barplot with the relative abundance per phylum per response group. Response groups are labeled with numbers and correspond to the groups in A, 1 is the bottom group, 4 is the top group, the rest is also shown in order.

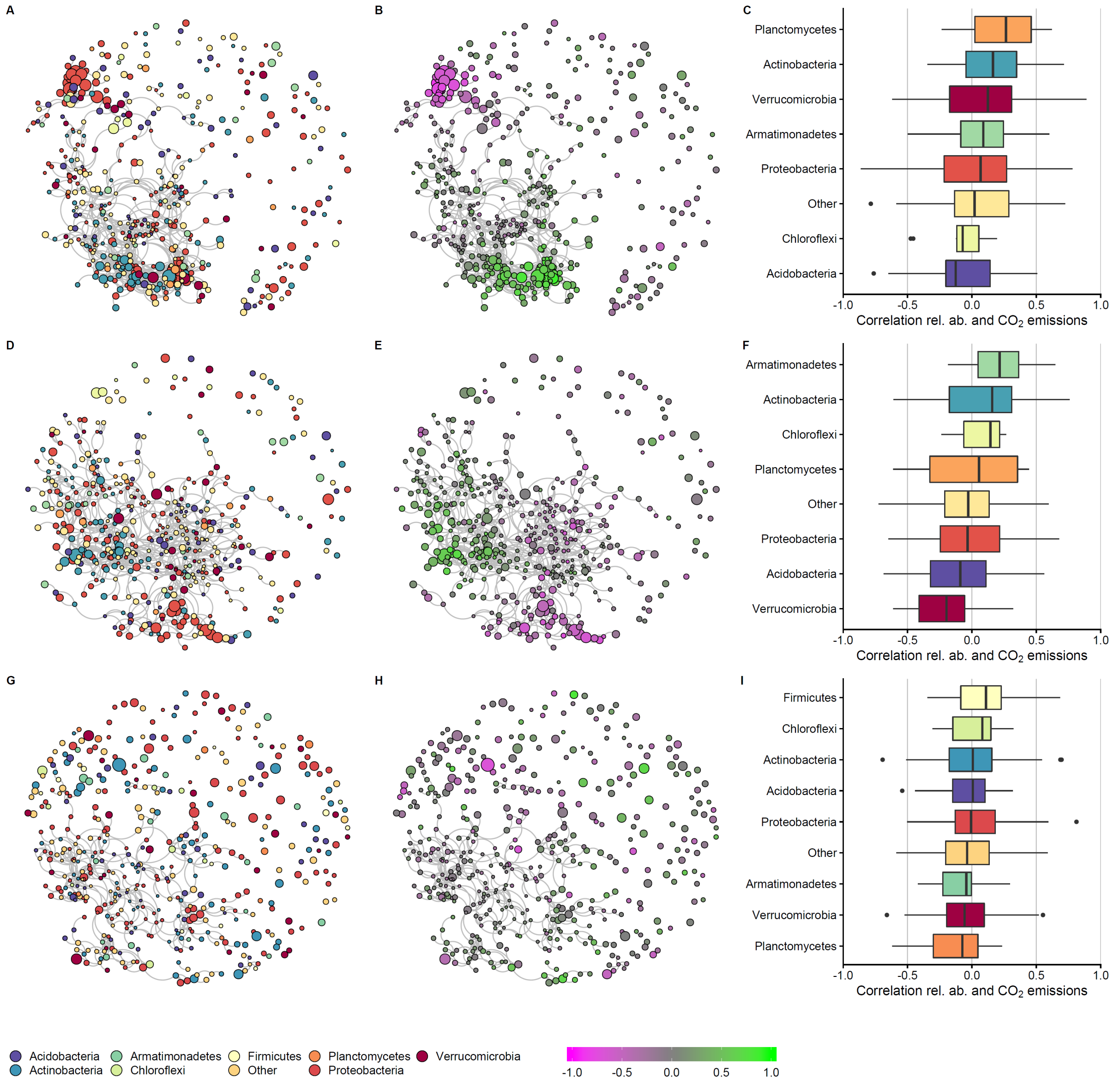

Fig. S14 Co-response networks of the 16S OTUs at DNA level and correlation between relative abundance and CO_2 emissions. A), B), C) show experiment 1, single treatments. D), E), F) show experiment 2, single treatments followed by a drouht treatment. G), H), I), show experiment 3, single treatments followed by a freezing-thawing treatment. A), D), G) co-response network with phylum overlay, legend below panel G). Node sizes are indicative for the mean relative abundance. B), E), H) show the co-response network with correlation between relative abundance and CO_2 emissions, legend is shown below panel H). C), F), I), shows boxplots with the correlations per OTU per phylum.

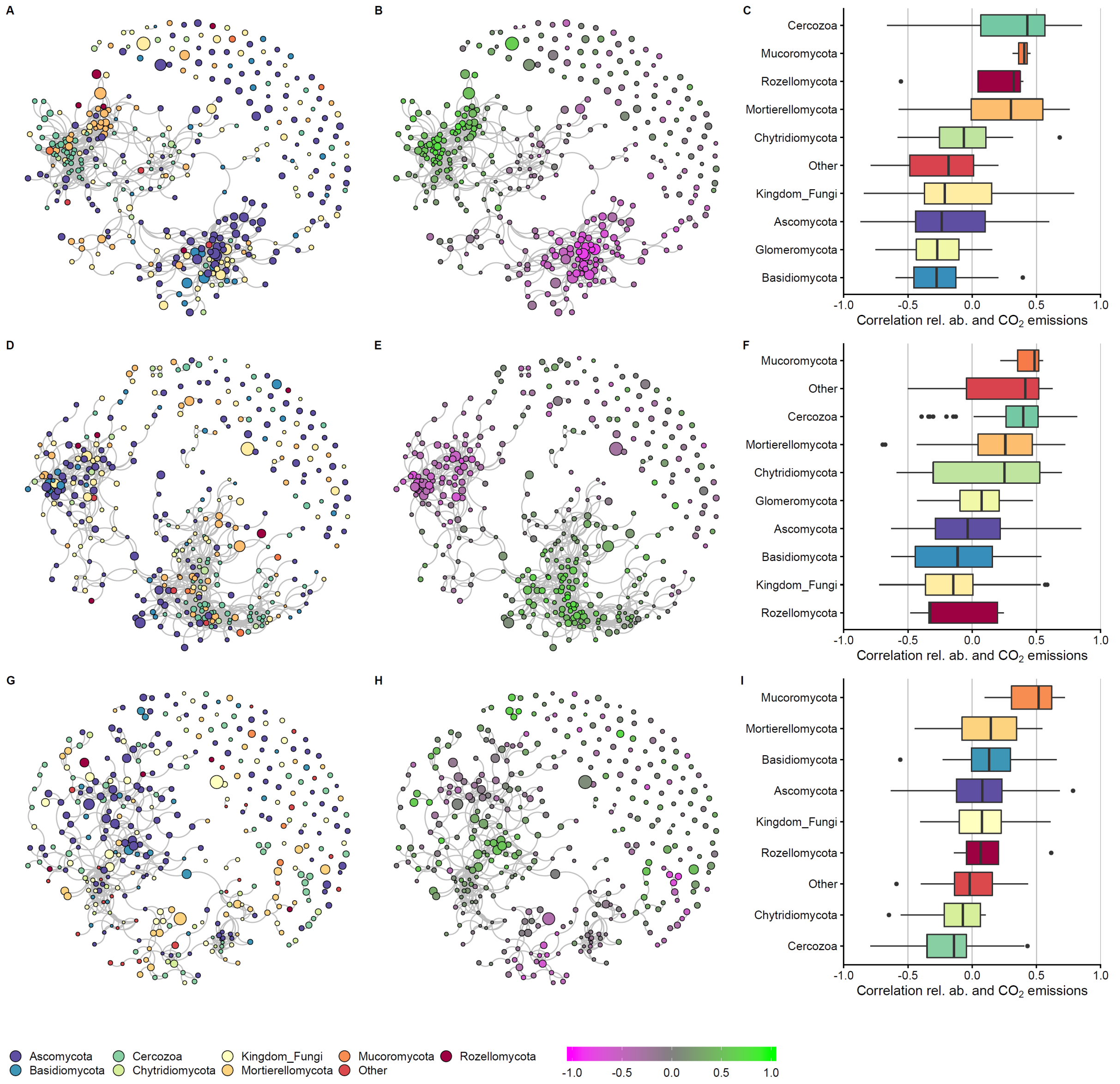

Fig. S15 Co-response networks of the ITS OTUs at DNA level and correlation between relative abundance and CO_2 emissions. A), B), C) show experiment 1, single treatments. D), E), F) show experiment 2, single treatments followed by a drouht treatment. G), H), I), show experiment 3, single treatments followed by a freeze-thaw treatments. A), D), G) co-response network with phylum overlay, legend below panel G). Node sizes are indicative for the mean relative abundance. B), E), H) show the co-response network with correlation between relative abundance and CO_2 emissions, legend is shown below panel H). C), F), I), shows bowplots with the correlations per OTU per phylum.

Table S1 Summary table of 16S rRNA transcripts. One sample was excluded as the extraction of RNA failed.

Samples	Experiment	jar	treatment	block	#reads[1]	notes
ANNELEIN-01	exp 1	85	DW	1	12127	
ANNELEIN-21	exp 1	86	FT	1	16214	
87B116SV3V4	exp 1	87	21ºC	3	17307	
ANNELEIN-49	exp 1	88	12ºC	4	16597	
8916SV3V4	exp 1	89	21ºC	4	11731	
ANNELEIN-62	exp 1	90	DW	3	13882	
9116SV3V4	exp 1	91	FT	3	24196	
ANNELEIN-09	exp 1	92	12ºC	3	11508	
ANNELEIN-28	exp 1	93	FT	5	15421	
ANNELEIN-33	exp 1	94	DW	4	12555	
9516SV3V4	exp 1	95	FT	4	12121	
9616SV3V4	exp 1	96	21ºC	2	14028	
9716SV3V4	exp 1	97	21ºC	5	22164	
87G116SV3V4	exp 1	98	12ºC	2	24322	
ANNELEIN-26	exp 1	99	12ºC	1	11612	
ANNELEIN-24	exp 1	121	DW	2	23655	
ANNELEIN-64	exp 1	121	FT	2	13961	
ANNELEIN-19	exp 1	122	21ºC	1	17500	
ANNELEIN-17	exp 1	123	DW	5	17041	
ANNELEIN-57	exp 1	124	12ºC	5	*9951*	
12516SV3V4	exp 2	125	DW-DW	2	14738	
12616SV3V4	exp 2	126	DW-DW	5	14461	
12116SV3V4	exp 2	121	DW-DW	3	15958	
13416SV3V4	exp 2	134	FT-DW	1	13185	
13516SV3V4	exp 2	135	FT-DW	2	18939	
14716SV3V4	exp 2	147	FT-DW	4	26822	
14816SV3V4	exp 2	148	FT-DW	5	30316	
ANNELEIN-11	exp 2	155	21ºC-DW	2	12406	
ANNELEIN-12	exp 2	129	21ºC-DW	3	12560	
ANNELEIN-14	exp 2	157	DW-DW	4	13962	
ANNELEIN-16	exp 2	137	21ºC-DW	4	22432	
ANNELEIN-22	exp 2	131	12ºC-DW	1	12527	
ANNELEIN-23	exp 2	145	12ºC-DW	3	15278	
ANNELEIN-25	exp 2	159	12ºC-DW	5	15350	
ANNELEIN-41	exp 2	133	FT-DW	3	11953	
ANNELEIN-46	exp 2	136	12ºC-DW	2	13875	
ANNELEIN-54	exp 2	128	12ºC-DW	4	12721	
ANNELEIN-55	exp 2	139	DW-DW	1	12497	
ANNELEIN-56	exp 2	112	21ºC-DW	1	24993	
ANNELEIN-63	exp 2	126	21ºC-DW	5	*12302*	
11616SV3V4	exp 3	116	21ºC-FT	1	16385	
11916SV3V4	exp 3	119	FT-FT	3	12322	
12716SV3V4	exp 3	127	FT-FT	4	21221	
14316SV3V4	exp 3	143	12ºC-FT	4	30984	
14616SV3V4	exp 3	146	FT-FT	5	16806	
14916SV3V4	exp 3	149	12ºC-FT	2	25591	
15616SV3V4	exp 3	156	21ºC-FT	4	32866	
ANNELEIN-12	exp 3	124	21ºC-FT	5	7035	
ANNELEIN-15	exp 3	142	FT-FT	2	*9511*	
ANNELEIN-21	exp 3	130	12ºC-FT	5	12895	

ANNELEIN-27	exp 3	123	DW-FT	2	17981	
ANNELEIN-29	exp 3	115	DW-FT	4	21743	
ANNELEIN-30	exp 3	140	DW-FT	3	15250	
ANNELEIN-31	exp 3	144	12ºC-FT	1	14148	
ANNELEIN-32	exp 3	132	DW-FT	1	24244	
ANNELEIN-45	exp 3	154	12ºC-FT	3	12157	
ANNELEIN-47	exp 3	111	DW-FT	5	11122	
ANNELEIN-48	exp 3	114	21ºC-FT	3	23425	
ANNELEIN-61	exp 3	150	21ºC-FT	2	13361	
		152	FT-FT	1		extraction failed

[1]reads in italic and underlined. The rarefraction depth was determined by the number of reads in italic and underlined within the specific experiment (e.g. exp. 1, exp 2 or exp 3).

Table S2 Summary information of 16S rRNA gene amplicons. One sample was excluded as the extraction of DNA failed. One sample was excluded, because of too few reads.

samples	Experiment	jar	treatment	block	#reads[1]	notes
121D16SV3V4	exp 1	121	DW	2	4444	
121D16SV3V4	exp 1	121	FT	2	6478	
122D16SV3V4	exp 1	122	21ºC	1	7009	
123D16SV3V4	exp 1	123	DW	5	5832	
124D16SV3V4	exp 1	124	12ºC	5	8062	
85D16SV3V4	exp 1	85	DW	1	6831	
86D16SV3V4	exp 1	86	FT	1	13009	
87D16SV3V4	exp 1	87	21ºC	3	7993	
88D16SV3V4	exp 1	88	12ºC	4	1722	
89D16SV3V4	exp 1	89	21ºC	4	5151	
90D16SV3V4	exp 1	90	DW	3	3728	
91D16SV3V4	exp 1	91	FT	3	4211	
92D16SV3V4	exp 1	92	12ºC	3	15953	
93D16SV3V4	exp 1	93	FT	5	5214	
94D16SV3V4	exp 1	94	DW	4	*1393*	
95D16SV3V4	exp 1	95	FT	4	4515	
96D16SV3V4	exp 1	96	21ºC	2	3627	
97D16SV3V4	exp 1	97	21ºC	5	2469	
98D16SV3V4	exp 1	98	12ºC	2	3602	
99D16SV3V4	exp 1	99	12ºC	1	11250	
125D16SV3V4	exp 2	125	DW-DW	2	*993*	
126D16SV3V4	exp 2	126	DW-DW	5	1499	
129D16SV3V4	exp 2	129	21ºC-DW	3	1559	
112D16SV3V4	exp 2	112	21ºC-DW	1	12332	
121D16SV3V4	exp 2	121	DW-DW	3	7040	
126D16SV3V4	exp 2	126	21ºC-DW	5	3907	
128D16SV3V4	exp 2	128	12ºC-DW	4	6124	
131D16SV3V4	exp 2	131	12ºC-DW	1	7344	
133D16SV3V4	exp 2	133	FT-DW	3	1316	
134D16SV3V4	exp 2	134	FT-DW	1	7123	
135D16SV3V4	exp 2	135	FT-DW	2	7276	
136D16SV3V4	exp 2	136	12ºC-DW	2	1588	
137D16SV3V4	exp 2	137	21ºC-DW	4	9221	
139D16SV3V4	exp 2	139	DW-DW	1	1211	
145D16SV3V4	exp 2	145	12ºC-DW	3	259	too few reads
147D16SV3V4	exp 2	147	FT-DW	4	2156	
148D16SV3V4	exp 2	148	FT-DW	5	3316	

155D16SV3V4	exp 2	155	21ºC-DW	2	1978	
157D16SV3V4	exp 2	157	DW-DW	4	6360	
159D16SV3V4	exp 2	159	12ºC-DW	5	1146	
111D16SV3V4	exp 3	111	DW-FT	5	*1236*	
114D16SV3V4	exp 3	114	21ºC-FT	3	8305	
115D16SV3V4	exp 3	115	DW-FT	4	2233	
116D16SV3V4	exp 3	116	21ºC-FT	1	12908	
119D16SV3V4	exp 3	119	FT-FT	3	2836	
123D16SV3V4	exp 3	123	DW-FT	2	2153	
124D16SV3V4	exp 3	124	21ºC-FT	5	12781	
127D16SV3V4	exp 3	127	FT-FT	4	12930	
130D16SV3V4	exp 3	130	12ºC-FT	5	11902	
132D16SV3V4	exp 3	132	DW-FT	1	2491	
140D16SV3V4	exp 3	140	DW-FT	3	4507	
142D16SV3V4	exp 3	142	FT-FT	2	1886	
143D16SV3V4	exp 3	143	12ºC-FT	4	5733	
144D16SV3V4	exp 3	144	12ºC-FT	1	2864	
146D16SV3V4	exp 3	146	FT-FT	5	3339	
149D16SV3V4	exp 3	149	12ºC-FT	2	5400	
150D16SV3V4	exp 3	150	21ºC-FT	2	9868	
154D16SV3V4	exp 3	154	12ºC-FT	3	7840	
156D16SV3V4	exp 3	156	21ºC-FT	4	2578	
		152	FT-FT	1		Extraction failed

[1]reads in italic and underlined. The rarefraction depth was determined by the number of reads in italic and underlined within the specific experiment (e.g. exp. 1, exp 2 or exp 3).

Table S3 Summary information of ITS transcripts. One sample was excluded as extraction of RNA failed.

Samples	Experiment	jar	treatment	block	#reads[1]	notes
87B1ITS	exp 1	87	21ºC	3	*43808*	
87G1ITS	exp 1	98	12ºC	2	46085	
99ITS	exp 1	99	12ºC	1	54218	
85ITS	exp 1	85	DW	1	54871	
97ITS	exp 1	97	21ºC	5	54897	
95ITS	exp 1	95	FT	4	56656	
91E1ITS	exp 1	91	FT	3	57575	
86ITS	exp 1	86	FT	1	58505	
92ITS	exp 1	92	12ºC	3	58950	
90ITS	exp 1	90	DW	3	61160	
88ITS	exp 1	88	12ºC	4	64041	
123ITS	exp 1	123	DW	5	67095	
89ITS	exp 1	89	21ºC	4	67349	
121ITS	exp 1	121	DW	2	68542	
94A4ITS	exp 1	94	DW	4	73545	
121ITS	exp 1	121	FT	2	75374	
124ITS	exp 1	124	12ºC	5	78584	
96ITS	exp 1	96	21ºC	2	80832	
122ITS	exp 1	122	21ºC	1	84052	
93ITS	exp 1	93	FT	5	84315	
125ITS	exp 2	125	DW-DW	2	64591	
126ITS	exp 2	126	DW-DW	5	69839	
169ITS	exp 2	129	21ºC-DW	3	37166	
112ITS	exp 2	112	21ºC-DW	1	73254	

121ITS	exp 2	121	DW-DW	3	51821	
126ITS	exp 2	126	21ºC-DW	5	72121	
128ITS	exp 2	128	12ºC-DW	4	48482	
151ITS	exp 2	131	12ºC-DW	1	49123	
133ITS	exp 2	133	FT-DW	3	45196	
134ITS	exp 2	134	FT-DW	1	48821	
135ITS	exp 2	135	FT-DW	2	46848	
136ITS	exp 2	136	12ºC-DW	2	*31222*	
137ITS	exp 2	137	21ºC-DW	4	58349	
139ITS	exp 2	139	DW-DW	1	38456	
145ITS	exp 2	145	12ºC-DW	3	52733	
147ITS	exp 2	147	FT-DW	4	51668	
148ITS	exp 2	148	FT-DW	5	63177	
155ITS	exp 2	155	21ºC-DW	2	34026	
157ITS	exp 2	157	DW-DW	4	44827	
159ITS	exp 2	159	12ºC-DW	5	62464	
111ITS	exp 3	111	DW-FT	5	53144	
114ITS	exp 3	114	21ºC-FT	3	50555	
115ITS	exp 3	115	DW-FT	4	58937	
116ITS	exp 3	116	21ºC-FT	1	66523	
119ITS	exp 3	119	FT-FT	3	91976	
123ITS	exp 3	123	DW-FT	2	*47409*	
124ITS	exp 3	124	21ºC-FT	5	49622	
127ITS	exp 3	127	FT-FT	4	69343	
130ITS	exp 3	130	12ºC-FT	5	72387	
132ITS	exp 3	132	DW-FT	1	48503	
140ITS	exp 3	140	DW-FT	3	80918	
142ITS	exp 3	142	FT-FT	2	70288	
143ITS	exp 3	143	12ºC-FT	4	60938	
144ITS	exp 3	144	12ºC-FT	1	48617	
146ITS	exp 3	146	FT-FT	5	83555	
149ITS	exp 3	149	12ºC-FT	2	56948	
150ITS	exp 3	150	21ºC-FT	2	75872	
154ITS	exp 3	154	12ºC-FT	3	59064	
156ITS	exp 3	156	21ºC-FT	4	81723	
		152	FT-FT	1		extraction failed

[1]reads in italic and underlined. The rarefraction depth was determined by the number of reads in italic and underlined within the specific experiment (e.g. exp. 1, exp 2 or exp 3).

Table S4 Summary information of ITS rRNA gene amplicons. One sample was excluded as extraction of DNA failed.

Samples	Experiment	jar	treatment	block	#reads[1]	notes
85DITS	exp 1	85	DW	1	38583	
86DITS	exp 1	86	FT	1	40634	
87DITS	exp 1	87	21ºC	3	48703	
88DITS	exp 1	88	12ºC	4	73634	
89DITS	exp 1	89	21ºC	4	41252	
90DITS	exp 1	90	DW	3	41284	
91DITS	exp 1	91	FT	3	43162	
92DITS	exp 1	92	12ºC	3	53437	
93DITS	exp 1	93	FT	5	62781	
94DITS	exp 1	94	DW	4	65077	
95DITS	exp 1	95	FT	4	53000	

96DITS	exp 1	96	21ºC	2	65190	
97DITS	exp 1	97	21ºC	5	43755	
98DITS	exp 1	98	12ºC	2	53988	
99DITS	exp 1	99	12ºC	1	44253	
121DITS	exp 1	121	DW	2	*34147*	
121DITS	exp 1	121	FT	2	43342	
122DITS	exp 1	122	21ºC	1	69062	
123DITS	exp 1	123	DW	5	68632	
124DITS	exp 1	124	12ºC	5	35471	
125DITS	exp 2	125	DW-DW	2	*27405*	
126DITS	exp 2	126	DW-DW	5	46875	
129DITS	exp 2	129	21ºC-DW	3	64838	
112DITS	exp 2	112	21ºC-DW	1	50390	
121DITS	exp 2	121	DW-DW	3	38812	
126DITS	exp 2	126	21ºC-DW	5	59064	
128DITS	exp 2	128	12ºC-DW	4	67879	
131DITS	exp 2	131	12ºC-DW	1	50877	
133DITS	exp 2	133	FT-DW	3	42426	
134DITS	exp 2	134	FT-DW	1	33015	
135DITS	exp 2	135	FT-DW	2	33621	
136DITS	exp 2	136	12ºC-DW	2	38061	
137DITS	exp 2	137	21ºC-DW	4	49652	
139DITS	exp 2	139	DW-DW	1	60055	
145DITS	exp 2	145	12ºC-DW	3	61702	
147DITS	exp 2	147	FT-DW	4	48916	
148DITS	exp 2	148	FT-DW	5	47726	
155DITS	exp 2	155	21ºC-DW	2	55698	
157DITS	exp 2	157	DW-DW	4	41167	
159DITS	exp 2	159	12ºC-DW	5	55751	
111DITS	exp 3	111	DW-FT	5	55505	
114DITS	exp 3	114	21ºC-FT	3	*27217*	
115DITS	exp 3	115	DW-FT	4	56027	
116DITS	exp 3	116	21ºC-FT	1	62409	
119DITS	exp 3	119	FT-FT	3	54157	
123DITS	exp 3	123	DW-FT	2	43212	
124DITS	exp 3	124	21ºC-FT	5	36133	
127DITS	exp 3	127	FT-FT	4	60891	
130DITS	exp 3	130	12ºC-FT	5	73528	
132DITS	exp 3	132	DW-FT	1	52195	
140DITS	exp 3	140	DW-FT	3	56349	
142DITS	exp 3	142	FT-FT	2	43386	
143DITS	exp 3	143	12ºC-FT	4	56935	
144DITS	exp 3	144	12ºC-FT	1	50280	
146DITS	exp 3	146	FT-FT	5	43271	
149DITS	exp 3	149	12ºC-FT	2	49264	
150DITS	exp 3	150	21ºC-FT	2	59390	
154DITS	exp 3	154	12ºC-FT	3	36957	
156DITS	exp 3	156	21ºC-FT	4	64256	
		152	FT-FT	1		Extraction failed

[1]reads in italic and underlined. The rarefraction depth was determined by the number of reads in italic and underlined within the specific experiment (e.g. exp. 1, exp 2 or exp 3).

Table S5. Results of permutational multivariate analysis of variance using distance matrices using Adonis function on Bray-Curtis dissimilarities of 16S OTUs of cDNA and DNA. Exp.1, Exp.2, Exp.3 refer to the three experiments (See Fig. 1 for description of experiments). Bold P values indicate p-value < 0.05. sum sq is sum of squares.

Molecule	Experiment		DF	Sum sq	R2	F	P
cDNA	Exp.1	Treatment	3	0.54	0.30	2.29	**0.002**
		Residuals	16	1.26	0.70		
	Exp.2	Treatment	3	0.28	0.18	1.15	0.08
		Residuals	16	1.29	0.82		
	Exp.3	Treatment	3	0.43	0.22	1.41	**0.009**
		Residuals	15	1.51	0.82		
DNA	Exp.1	Treatment	3	0.69	0.27	1.93	**0.003**
		Residuals	16	1.91	0.73		
	Exp.2	Treatment	3	0.2992	0.09	0.50	0.86
		Residuals	15	2.9677	0.91		
	Exp.3	Treatment	3	1.01	0.35	2.65	**0.012**
		Residuals	15	1.91	0.65		

Table S6. Results of permutational multivariate analysis of variance using distance matrices using Adonis function on Bray-Curtis dissimilarities of ITS OTUs of cDNA and DNA. Exp.1, Exp.2, Exp.3 refer to the three experiments (See Fig. 1 for description of experiments). Bold P values indicate p-value < 0.05. sum sq is sum of squares.

Molecule	Experiment		DF	Sum sq	R2	F	P
cDNA	Exp.1	Treatment	3	0.375	0.21	1.34	**<0.001**
		Residuals	16	1.496	0.80		
	Exp.2	Treatment	3	0.26	0.13	0.78	**0.005**
		Residuals	16	1.78	0.87		
	Exp.3	Treatment	3	0.49	0.27	1.89	**<0.001**
		Residuals	15	1.29	0.73		
DNA	Exp.1	Treatment	3	0.32	0.18	1.2	**0.002**
		Residuals	16	1.44	0.82		
	Exp.2	Treatment	3	0.35	0.17	1.08	**0.002**
		Residuals	16	1.75	0.83		
	Exp.3	Treatment	3	0.32	0.18	1.08	**0.003**
		Residuals	15	1.48	0.82		
